# Absence of association of a single-nucleotide polymorphism in the *TERT-CLPTM1L* locus with age-related phenotypes in a large multicohort study: the HALCyon programme

**DOI:** 10.1111/j.1474-9726.2011.00687.x

**Published:** 2011-06

**Authors:** Tamuno Alfred, Yoav Ben-Shlomo, Rachel Cooper, Rebecca Hardy, Cyrus Cooper, Ian J Deary, Jane Elliott, David Gunnell, Sarah E Harris, Mika Kivimaki, Meena Kumari, Richard M Martin, Chris Power, Avan Aihie Sayer, John M Starr, Diana Kuh, Ian N M Day, the HALCyon Study Team

**Affiliations:** 1 School of Social and Community Medicine, University of BristolCanynge Hall, 39 Whatley Road, Bristol BS8 2PS, UK; 2 MRC Unit for Lifelong Health and Ageing and Division of Population Health, University College London33 Bedford Place London, WC1B 5JU, London, UK; 3 MRC Lifecourse Epidemiology Unit, University of Southampton, Southampton General Hospital SouthamptonSO16 6YD, UK; 4 Institute of Musculoskeletal Sciences, University of OxfordWindmill Road, Oxford, OX3 7LD, UK; 5 Centre for Cognitive Ageing and Cognitive Epidemiology, University of Edinburgh7 George Square Edinburgh, EH8 9JZ, UK; 6 Department of Psychology, University of Edinburgh7 George Square, Edinburgh, EH8 9JZ, UK; 7 Centre for Longitudinal Studies, Institute of EducationBedford Group, 20 Bedford Way, London WC1H 0AL, UK; 8 Medical Genetics Section, University of Edinburgh, Western General HospitalCrewe Road, Edinburgh, EH4 2XU, UK; 9 Department of Epidemiology and Public HealthUCL, 1 - 19 Torrington Place, London WC1E 6BT, UK; 10 MRC Centre for Causal Analyses in Translational Epidemiology, University of BristolOakfield House, Oakfield Grove, Bristol, BS8 2BN, UK; 11 MRC Centre of Epidemiology for Child Health /Centre for Paediatric Epidemiology and Biostatistics, UCL Institute of Child Health30 Guilford Street, London, WC1N 1EH, UK; 12 Academic Geriatric Medicine, University of Southampton, Southampton General HospitalSouthampton, SO16 6YD, UK; 13 Geriatric Medicine Unit, Royal Victoria Hospital, University of EdinburghCraigleith Road, Edinburgh, EH4 2DN, UK

**Keywords:** aging, cognition, middle-aged, physical, telomere

## Abstract

Several age-related traits are associated with shorter telomeres, the structures that cap the end of linear chromosomes. A common polymorphism near the telomere maintenance gene *TERT* has been associated with several cancers, but relationships with other aging traits such as physical capability have not been reported. As part of the Healthy Ageing across the Life Course (HALCyon) collaborative research programme, men and women aged between 44 and 90 years from nine UK cohorts were genotyped for the single-nucleotide polymorphism (SNP) rs401681. We then investigated relationships between the SNP and 30 age-related phenotypes, including cognitive and physical capability, blood lipid levels and lung function, pooling within-study genotypic effects in meta-analyses. No significant associations were found between the SNP and any of the cognitive performance tests (e.g. pooled beta per T allele for word recall *z*-score = 0.02, 95% CI: −0.01 to 0.04, *P*-value = 0.12, *n* = 18 737), physical performance tests (e.g. pooled beta for grip strength = −0.02, 95% CI: −0.045 to 0.006, *P*-value = 0.14, *n* = 11 711), blood pressure, lung function or blood test measures. Similarly, no differences in observations were found when considering follow-up measures of cognitive or physical performance after adjusting for its measure at an earlier assessment. The lack of associations between SNP rs401681 and a wide range of age-related phenotypes investigated in this large multicohort study suggests that while this SNP may be associated with cancer, it is not an important contributor to other markers of aging.

## Introduction

Aging is caused by the accumulation of molecular and cellular damage over time resulting in frailty and disease (Kirkwood, 2008). One likely source is from damage to DNA reflected in telomere shortening (Chan & Blackburn, 2004; Kirkwood, 2008). Telomeres are formed from a repetitive DNA sequence (TTAGGG 5′ to 3′) and a variety of proteins, and are located on the end of linear chromosomes. They protect against the loss of genetic material during cell division (Calado & Young, 2009). Human telomerase is an enzyme composed of two protein components: telomerase reverse transcriptase (encoded by the *TERT* gene) and dyskerin (encoded by *DKC1*), and a RNA component (encoded by *TERC*) that elongates telomeres by adding the telomeric repeats to the chromosome ends (Blasco, 2005; Cohen *et al.*, 2007; Calado & Young, 2009). However, owing to low telomerase activity in most normal human somatic cells (Blasco, 2005), telomeres undergo attrition with losses in the range of 20–70 bp per year estimated in adults (Benetos *et al.*, 2001; Canela *et al.*, 2007; Codd *et al.*, 2010). Shorter telomere length has been associated with several age-related conditions (Aviv, 2006), such as hypertension (Huda *et al.*, 2007), coronary heart disease (Brouilette *et al.*, 2007), dementia (Martin-Ruiz *et al.*, 2006), premature aging syndromes (Blasco, 2005) and mortality (Bakaysa *et al.*, 2007). Although it remains unclear whether telomere shortening is a cause or simply a consequence of aging (Calado & Young, 2009), these associations have led to the widespread notion that telomere shortening represents a mechanism for aging in general.

While it has been suggested that oxidative stress and inflammation are contributors to telomere loss (von Zglinicki, 2002; Valdes *et al.*, 2005; Ornish *et al.*, 2008; Starr *et al.*, 2008), telomere length is highly heritable with family studies providing estimates between 35% and 80% (Slagboom *et al.*, 1994; Bischoff *et al.*, 2005; Vasa-Nicotera *et al.*, 2005; Bakaysa *et al.*, 2007). As a putative polygenic trait, several loci have been implicated (Vasa-Nicotera *et al.*, 2005; Andrew *et al.*, 2006; Mangino *et al.*, 2008, 2009; Starr *et al.*, 2008; Levy *et al.*, 2010), with some including genes known to be directly involved in telomere maintenance in humans (Codd *et al.*, 2010). In addition to telomerase activity (Ludlow *et al.*, 2008) and telomere length (Matsubara *et al.*, 2006a, b), single-nucleotide polymorphisms (SNPs) located in the *TERT*-*CLPTM1L* locus have been associated with exceptional longevity (Atzmon *et al.*, 2010), coronary artery disease (Matsubara *et al.*, 2006a), idiopathic pulmonary fibrosis (Mushiroda *et al.*, 2008), glioma (Shete *et al.*, 2009), red blood cell count (Kamatani *et al.*, 2010), survival in patients with lung cancer (Catarino *et al.*, 2010) and several cancers (Ruiz-Llorente *et al.*, 2007; McKay *et al.*, 2008; Andrew *et al.*, 2009; Broderick *et al.*, 2009; Choi *et al.*, 2009; Hosgood *et al.*, 2009; Landi *et al.*, 2009; Van Dyke *et al.*, 2009; Zienolddiny *et al.*, 2009; Hsiung *et al.*, 2010; Johnatty *et al.*, 2010; Prescott *et al.*, 2010; Shen *et al.*, 2010; Turnbull *et al.*, 2010; Wang *et al.*, 2010). Specifically, relationships have been reported between the C allele of a common, intronic polymorphism in *CLPTM1L* (rs401681) and shorter telomere length in elderly females (Rafnar *et al.*, 2009), increased PSA levels (Gudmundsson *et al.*, 2010), increased basal cell carcinoma (Rafnar *et al.*, 2009; Stacey *et al.*, 2009), prostate (Rafnar *et al.*, 2009), cervical (Rafnar *et al.*, 2009), bladder (Rothman *et al.*, 2010) and lung (Wang *et al.*, 2008; Rafnar *et al.*, 2009; Kohno *et al.*, 2010; Miki *et al.*, 2010) cancer risk and a reduced risk of melanoma (Stacey *et al.*, 2009) and pancreatic (Petersen *et al.*, 2010) cancer, although there is a lack of association with breast cancer (Rafnar *et al.*, 2009; Pooley *et al.*, 2010). Over-expression of CLPTM1L (cisplatinum resistance related protein) has been associated with apoptosis (Yamamoto *et al.*, 2001). It should be noted that cancer incidence increases steeply with age (Nordling, 1953; DePinho, 2000; Cancer Research UK, 2010).

We therefore hypothesized that polymorphisms in the *TERT-CLPTM1L* locus could be related to other age-related phenotypes, such as lower physical and cognitive capability, traits that are known to have a genetic component (McClearn *et al.*, 1997; Tiainen *et al.*, 2004; Volk *et al.*, 2006; Matteini *et al.*, 2010). To investigate this, we analysed data from 25 774 participants aged between 44 and 90 from nine UK cohorts as part of the Healthy Ageing across the Life Course (HALCyon; http://www.halcyon.ac.uk/) collaborative research programme. The HALCyon programme aims to understand three components of healthy aging: (i) physical and cognitive capability; (ii) psychological and social wellbeing; and (iii) the underlying biology. The well-established cohorts are all appropriate for investigating our hypothesis, as all studies measured markers of physical or cognitive capability or biological functioning in older adults. In this exploratory study, we genotyped participants for the SNP rs401681 and conducted analyses within the cohorts as well as meta-analyses to assess associations between genotype and 30 age-related phenotypes including word recall score, grip strength, lung function and cardiometabolic biomarkers. We believe this to be the first report of an investigation between a SNP in this locus and such traits.

## Results

### Cohort summaries and genotyping quality

Successful genotyping for SNP rs401681 and relevant phenotypes were available for a total of 25 774 adults aged between 44 and 90 years old (Table 1). The quality of the genotyping was good and consistent across all nine studies, with similar frequencies, call rates exceeding 97% and the HWE condition being met (*P*-value ≥0.3).

**Table 1:** Summary of sex, age and genotype frequencies by cohort

Cohort	Male (%)	Age* in years, median (range)	C/C *n* (%)	C/T *n* (%)	T/T *n* (%)	Total (*n*)
Boyd Orr	46	70 (64 to 82)	227 (32.7)	341 (49.1)	127 (18.3)	695
CaPS	100	57 (47 to 67)	414 (29.9)	698 (50.5)	271 (19.6)	1383
ELSA	46	65 (52 to 90+)	1672 (30.7)	2709 (49.7)	1067 (19.6)	5448
HAS	61	67 (63 to 73)	159 (30.2)	267 (50.7)	101 (19.2)	527
HCS	53	66 (59 to 73)	905 (32.3)	1400 (49.9)	501 (17.9)	2806
LBC1921	42	79 (77 to 80)	178 (34.4)	249 (48.2)	90 (17.4)	517
NCDS	51	44 (44 to 45)	2317 (31.6)	3587 (49.0)	1417 (19.4)	7321
NSHD	50	53	780 (30.3)	1277 (49.6)	519 (20.1)	2576
Whitehall II	76	59 (50 to 73)	1467 (32.6)	2200 (48.9)	834 (18.5)	4501
Total	57	56 (44 to 90+)	8119 (31.5)	12 728 (49.4)	4927 (19.1)	25 774

CaPS, Caerphilly Prospective Study; ELSA, English Longitudinal Study of Ageing; HAS, Hertfordshire Ageing Study; HCS, Hertfordshire Cohort Study; NCDS, National Child Development Study; NSHD, National Survey of Health and Development.

* Age at phase from which the majority of variables are taken, i.e. Boyd Orr: III; CaPS: II; ELSA: II; HAS: I; HCS: I; LBC1921: I; NCDS: Biomedical Survey (2002); NSHD: 1999 Collection; Whitehall II: VII.

### Associations between genotype and phenotypes

Minor allele frequencies (MAF) were tabulated by sex, 5-year age bands (Table S1 in Supporting Information), cigarette smoking status and physical activity (data not shown) to identify possible confounders of genotype effect on the outcome variables; no associations were observed with the MAF so such variables were not included in the regression models.

Table 2 shows there was no evidence of any associations between the genotype and any of the cognitive test scores in the meta-analyses (all *P*-values >0.1). Results for the genotypic associations were similar in the within-study analyses investigating cognition scores in later phases adjusting for score at an earlier phase (data not shown). Significant heterogeneity between subgroups was only observed for AH4 analysed by sex (Fig. S2b in Supporting Information), which was mainly driven by Whitehall II (interaction *P*-value = 0.0027), although this observation was not seen for any other phenotype.

**Table 2:** Cognitive capability scores by genotype and cohort

Variable	Cohort	C/C Mean (SD) [*n*]	C/T Mean (SD) [*n*]	T/T Mean (SD) [*n*]	Total Mean (SD) [*n*]	*b* (95% CI)*	*P*-value	Het *P*-value
NART	CaPS	24.2 (12.0) [371]	23.8 (11.8) [628]	23.0 (11.2) [238]	23.7 (11.8) [1237]	−0.05 (−0.13 to 0.03)	0.22	0.20
	LBC1921	34.6 (8.0) [178]	34.3 (8.5) [247]	33.7 (8.1) [90]	34.3 (8.2) [515]	−0.05 (−0.17 to 0.08)	0.44	
	NSHD	34.1 (9.8) [734]	34.5 (9.4) [1217]	34.6 (9.4) [501]	34.4 (9.5) [2452]	0.03 (−0.02 to 0.09)	0.27	
	Pooled	[1283]	[2092]	[829]	[4204]	−0.010 (−0.070 to 0.049)	0.73	
AH4	CaPS	25.6 (11.0) [373]	25.3 (11.0) [622]	24.5 (10.4) [238]	25.2 (10.9) [1233]	−0.05 (−0.13 to 0.03)	0.21	0.31
	HAS	20.8 (9.1) [153]	23.0 (9.2) [260]	21.5 (8.9) [97]	22.1 (9.2) [510]	0.06 (−0.06 to 0.19)	0.33	
	Whitehall II	45.3 (10.4) [1440]	45.1 (10.4) [2173]	45.1 (10.3) [818]	45.2 (10.4) [4431]	−0.01 (−0.05 to 0.04)	0.76	
	Pooled	[1966]	[3055]	[1153]	[6174]	−0.010 (−0.052 to 0.032)	0.65	
Mill Hill vocabulary	HAS	18.7 (4.0) [141]	18.3 (4.4) [261]	17.7 (3.9) [92]	18.3 (4.2) [494]	−0.12 (−0.25 to 0.01)	0.07	0.09
	Whitehall II	26.0 (3.0) [1369]	25.7 (3.5) [2123]	26.1 (2.6) [746]	25.9 (3.2) [4238]	−0.00 (−0.05 to 0.04)	0.95	
	Pooled	[1510]	[2384]	[838]	[4732]	−0.044 (−0.156 to 0.067)	0.44	
Word recall – 10 words	ELSA	5.1 (1.7) [1665]	5.1 (1.7) [2704]	5.1 (1.8) [1064]	5.1 (1.7) [5433]	−0.00 (−0.04 to 0.04)	0.94	0.23
Word recall – 10 words	NCDS	6.0 (1.5) [2027]	6.0 (1.5) [3117]	6.1 (1.4) [1226]	6.0 (1.5) [6370]	0.01 (−0.03 to 0.04)	0.66	
Word recall – 45 words	NSHD	23.7 (6.4) [757]	24.1 (6.2) [1242]	24.0 (6.3) [509]	24.0 (6.3) [2508]	0.03 (−0.03 to 0.08)	0.36	
Word recall – 20 words	Whitehall II	6.8 (2.4) [1439]	6.9 (2.4) [2170]	7.1 (2.4) [817]	6.9 (2.4) [4426]	0.05 (0.01 to 0.10)	0.0107	
	Pooled	[5888]	[9233]	[3616]	[18 737]	0.020 (−0.005 to 0.045)	0.12	
Logical memory	LBC1921	32.6 (13.8) [177]	31.4 (12.8) [248]	31.3 (11.7) [90]	31.8 (13.0) [515]	−0.057 (−0.181 to 0.066)	0.36	0.57
Phonemic fluency – 3 letters	LBC1921	40.2 (12.2) [175]	40.2 (12.4) [248]	39.7 (12.2) [90]	40.1 (12.3) [513]	−0.02 (−0.14 to 0.11)	0.79	
Phonemic fluency – 1 letter	Whitehall II	15.8 (4.2) [1436]	16.0 (4.2) [2162]	16.0 (4.0) [814]	16.0 (4.1) [4412]	0.02 (−0.02 to 0.06)	0.32	
	Pooled	[1611]	[2410]	[904]	[4925]	0.018 (−0.022 to 0.057)	0.39	
Semantic fluency	CaPS	16.6 (4.9) [373]	16.4 (4.7) [632]	16.6 (4.6) [241]	16.5 (4.8) [1246]	−0.00 (−0.08 to 0.08)	0.94	0.42
	ELSA	20.4 (6.4) [1668]	20.3 (6.4) [2707]	20.1 (6.4) [1066]	20.3 (6.4) [5441]	−0.02 (−0.06 to 0.01)	0.20	
	NCDS	22.3 (6.4) [2042]	22.4 (6.2) [3137]	22.6 (6.4) [1233]	22.4 (6.3) [6412]	0.02 (−0.01 to 0.06)	0.21	
	NSHD	23.4 (7.1) [771]	23.8 (6.7) [1267]	23.6 (6.8) [517]	23.7 (6.9) [2555]	0.01 (−0.04 to 0.07)	0.63	
	Whitehall II	16.1 (3.7) [1438]	15.8 (3.8) [2168]	16.0 (3.6) [818]	15.9 (3.7) [4424]	−0.01 (−0.06 to 0.03)	0.49	
	Pooled	[6292]	[9911]	[3875]	[20 078]	−0.001 (−0.021 to 0.019)	0.91	
Search speed – 780 letters	ELSA	295 (72) [1550]	293 (82) [2614]	292 (59) [892]	293 (76) [5056]	−0.02 (−0.06 to 0.02)	0.35	0.16
Search speed – 780 letters	NCDS	328 (67) [1884]	326 (77) [3047]	325 (62) [1099]	326 (71) [6030]	−0.02 (−0.06 to 0.02)	0.27	
Search speed – 600 letters	NSHD	276 (65) [732]	278 (71) [1240]	282 (62) [485]	278 (67) [2457]	0.04 (−0.02 to 0.10)	0.16	
	Pooled	[4166]	[6901]	[2476]	[13 543]	−0.005 (−0.039 to 0.028)	0.76	
Raven’s Matrices	LBC1921	31.8 (8.6) [177]	30.9 (9.1) [247]	31.8 (8.2) [88]	31.3 (8.8) [512]	−0.015 (−0.140 to 0.109)	0.81	

CaPS: Phase III; NCDS: word recall, semantic fluency, search speed: Sweep 8 (2008).

Het, heterogeneity; CaPS, Caerphilly Prospective Study; ELSA, English Longitudinal Study of Ageing; HAS, Hertfordshire Ageing Study; HCS, Hertfordshire Cohort Study; NCDS, National Child Development Study; NSHD, National Survey of Health and Development.

* Beta coefficients per T allele based on *z*-scores.

No genotypic associations were observed for any of the physical capability measures in the meta-analyses (Table 3) (all *P*-values >0.1). In addition, there was no evidence of associations with grip strength in the follow-up phases adjusting for its measure at a previous phase.

**Table 3:** Physical capability by genotype and cohort

Variable	Cohort	C/C Mean (SD) [*n*]	C/T Mean (SD) [*n*]	T/T Mean (SD) [*n*]	Total Mean (SD) [*n*]	*b* (95% CI)*	*P*-value	Het *P*-value
Grip strength (kg)	ELSA	32.4 (11.8) [1661]	31.9 (11.4) [2680]	31.9 (11.6) [1056]	32.0 (11.6) [5397]	−0.03 (−0.06 to 0.01)	0.20	0.43
	HAS	32.2 (9.7) [157]	32.0 (10.3) [265]	31.3 (9.7) [100]	31.9 (10.0) [522]	−0.04 (−0.17 to 0.08)	0.48	
	HCS	36.2 (11.4) [905]	35.6 (10.9) [1399]	35.5 (10.8) [500]	35.8 (11.0) [2804]	−0.03 (−0.09 to 0.02)	0.22	
	LBC1921	27.7 (9.5) [177]	25.4 (8.6) [246]	26.9 (9.6) [90]	26.5 (9.1) [513]	−0.08 (−0.20 to 0.05)	0.21	
	NSHD	37.6 (14.3) [752]	38.1 (14.1) [1227]	38.2 (14.6) [496]	37.9 (14.3) [2475]	0.03 (−0.03 to 0.08)	0.38	
	Pooled	[3652]	[5817]	[2242]	[11 711]	−0.020 (−0.045 to 0.006)	0.14	
Log timed 3 m get up and go (s)	Boyd Orr	2.23 (0.29) [131]	2.24 (0.28) [188]	2.20 (0.23) [68]	2.23 (0.27) [387]	−0.03 (−0.17 to 0.11)	0.67	0.80
Log timed 3 m get up and go (s)	CaPS	2.35 (0.24) [236]	2.33 (0.25) [392]	2.32 (0.24) [165]	2.33 (0.25) [793]	−0.05 (−0.15 to 0.05)	0.35	
Log timed 2.44 m walk, s	ELSA	1.02 (0.36) [1044]	1.02 (0.36) [1729]	1.01 (0.37) [688]	1.02 (0.36) [3461]	−0.01 (−0.06 to 0.03)	0.55	
Log timed 3 m get up and go (s)	HAS	2.50 (0.31) [52]	2.51 (0.28) [110]	2.48 (0.18) [43]	2.51 (0.27) [205]	−0.03 (−0.24 to 0.17)	0.74	
Log timed 3 m get up and go (s)	HCS	2.37 (0.19) [706]	2.37 (0.19) [1073]	2.38 (0.19) [372]	2.37 (0.19) [2151]	0.03 (−0.03 to 0.09)	0.36	
Log timed 6 m walk (s)	LBC1921	1.50 (0.31) [177]	1.49 (0.27) [244]	1.49 (0.34) [89]	1.49 (0.30) [510]	−0.03 (−0.16 to 0.09)	0.60	
	Pooled	[2346]	[3736]	[1425]	[7507]	−0.009 (−0.041 to 0.024)	0.60	
Timed chair rises† – 5 rises	ELSA	9.2 (2.4) [1353]	9.5 (2.9) [2296]	9.3 (2.3) [807]	9.3 (2.7) [4456]	0.02 (−0.02 to 0.07)	0.27	0.17
Timed chair rises† – 5 rises	HAS	5.1 (1.1) [47]	5.4 (1.3) [101]	5.5 (1.1) [30]	5.3 (1.2) [178]	0.17 (−0.05 to 0.40)	0.13	
Timed chair rises† – 5 rises	HCS	6.3 (1.2) [440]	6.2 (1.4) [743]	6.2 (1.1) [211]	6.2 (1.3) [1394]	−0.04 (−0.12 to 0.04)	0.31	
Timed chair rises† – 10 rises	NSHD	5.0 (1.2) [674]	5.1 (1.5) [1162]	5.1 (1.2) [441]	5.1 (1.3) [2277]	0.05 (−0.01 to 0.11)	0.10	
	Pooled	[2514]	[4302]	[1489]	[8305]	0.024 (−0.023 to 0.070)	0.32	

		C/C *n* (%)‡	C/T *n* (%)‡	T/T *n* (%)‡	Total *n* (%)‡	OR (95% CI)§		
Balance <5 s – Flamingo	Boyd Orr	44 (33.6)	82 (43.9)	25 (36.8)	151 (39.1)	1.134 (0.846 to 1.519)	0.40	0.14
Balance <5 s – Flamingo	CaPS	94 (39.3)	150 (37.5)	49 (30.8)	293 (36.7)	0.841 (0.684 to 1.034)	0.10	
Balance <5 s – Tandem	ELSA	225 (13.5)	361 (13.3)	122 (11.4)	708 (13.0)	0.922 (0.823 to 1.033)	0.16	
Balance <5 s – Flamingo	HAS	18 (34.6)	37 (33.3)	11 (25.6)	66 (32.0)	0.818 (0.530 to 1.263)	0.37	
Balance <5 s – Flamingo	HCS	80 (16.0)	144 (18.7)	54 (20.7)	278 (18.1)	1.174 (0.973 to 1.417)	0.09	
Balance <5 s – One-legged	NSHD	34 (4.6)	45 (3.6)	21 (4.2)	100 (4.0)	0.931 (0.700 to 1.240)	0.63	
	Pooled¶	[495/3341]	[819/5423]	[282/2104]	[1596/10 868]	0.970 (0.864 to 1.089)	0.60	

CaPS: Phase V; HAS: balance, timed get up and go, chair rises: Phase II; HCS: balance, timed walk, chair rises: data from both phases.

Het, heterogeneity; CaPS, Caerphilly Prospective Study; ELSA, English Longitudinal Study of Ageing; HAS, Hertfordshire Ageing Study; HCS, Hertfordshire Cohort Study; NCDS, National Child Development Study; NSHD, National Survey of Health and Development.

* Beta coefficients based on *z*-scores per T allele.

† Reciprocal of time taken in seconds × 100.

‡ No. of participants unable to balance for at least 5 s (%).

§ OR per T allele.

¶ Pooled: [no. of participants unable to balance for at least 5 s/total no of. participants with relevant data].

Table 4 and Table S2 (Supporting Information) show no associations with BMI, waist–hip ratio (WHR) or the measures of biological function on the pooled analyses (all *P*-values >0.06).

**Table 4:** Anthropometry and biological function by genotype (pooled results)

Variable	Total	*b* (95% CI)*	*P*-value	Het *P*-value
BMI (kg m^−2^)	25 336	0.009 (−0.009 to 0.026)	0.33	0.81
Waist–hip ratio	22 134	−0.001 (−0.020 to 0.018)	0.93	0.72
Systolic blood pressure (mmHg)	25 030	−0.003 (−0.020 to 0.015)	0.77	0.67
Diastolic blood pressure (mmHg)	25 027	0.009 (−0.008 to 0.027)	0.29	0.75
Pulse rate (BPM)	18 355	0.007 (−0.018 to 0.033)	0.58	0.26
Forced vital capacity (L)	19 333	−0.001 (−0.027 to 0.025)	0.93	0.24
Forced expiratory volume (L)	19 338	−0.002 (−0.022 to 0.018)	0.86	0.47
Fibrinogen (g L^−^)	20 028	0.024 (−0.001 to 0.049)	0.06	0.23
Total cholesterol (mm)	23 098	0.020 (−0.003 to 0.043)	0.09	0.24
HDL cholesterol (mm)	23 616	0.014 (−0.018 to 0.045)	0.39	0.0207
Log triglycerides (mm)	24 279	−0.001 (−0.020 to 0.018)	0.91	0.39
LDL cholesterol (mm)	22 985	0.013 (−0.006 to 0.031)	0.18	0.67
Glucose†	22 572	0.001 (−0.017 to 0.020)	0.88	0.50

Het, heterogeneity.

* Beta coefficients per T allele based on *z*-scores.

† On scale 10 × (glucose in mm or HbA1c in %)^−2^.

There were no associations between genotype and MI, angina, diabetes or stroke on the pooled data (Table 5) (all *P*-values >0.1). There was some evidence of an association between the T allele and lower MI risk in Whitehall II (*P*-value = 0.005), although this was not seen in the other studies.

**Table 5:** Health history by genotype and cohort

Variable	Cohort	C/C *n* (%)*	C/T *n* (%)*	T/T *n* (%)*	Total *n* (%)*	OR (95% CI)†	*P*-value	Het *P*-value
MI	Boyd Orr	10 (4.4)	20 (5.9)	10 (7.9)	40 (5.8)	1.362 (0.866–2.142)	0.18	0.08
	CaPS	60 (14.7)	126 (18.3)	33 (12.4)	219 (16.1)	0.953 (0.774–1.173)	0.65	
	ELSA	87 (5.2)	146 (5.4)	52 (4.9)	285 (5.2)	0.976 (0.823–1.157)	0.78	
	HAS	15 (9.7)	21 (8.0)	10 (10.2)	46 (8.9)	1.004 (0.648–1.555)	0.99	
	Whitehall II	52 (3.8)	48 (2.3)	15 (1.9)	115 (2.7)	0.673 (0.511–0.888)	0.0050	
	Pooled	[224/3830]	[361/6053]	[120/2338]	[705/12 221]	0.936 (0.782–1.119)	0.47	
Angina	Boyd Orr	26 (13.1)	34 (11.9)	10 (9.7)	70 (11.9)	0.857 (0.597–1.231)	0.40	0.78
	CaPS	48 (20.6)	83 (21.1)	33 (21.2)	164 (20.9)	1.018 (0.796–1.303)	0.89	
	ELSA	160 (9.6)	237 (8.8)	94 (8.8)	491 (9.0)	0.950 (0.832–1.085)	0.45	
	HAS	21 (13.5)	27 (10.3)	15 (15.2)	63 (12.2)	1.036 (0.708–1.514)	0.86	
	Whitehall II	65 (4.7)	96 (4.6)	25 (3.2)	186 (4.4)	0.852 (0.689–1.054)	0.14	
	Pooled	[320/3649]	[477/5733]	[177/2212]	[974/11 594]	0.937 (0.852–1.031)	0.18	
Diabetes	Boyd Orr	12 (9.2)	17 (9.0)	10 (14.7)	39 (10.1)	1.286 (0.804–2.057)	0.29	0.88
	CaPS	31 (13.4)	49 (12.3)	25 (16.0)	105 (13.4)	1.099 (0.818–1.475)	0.53	
	ELSA	129 (7.7)	186 (6.9)	80 (7.5)	395 (7.3)	0.973 (0.840–1.126)	0.71	
	HAS	15 (10.1)	22 (8.9)	9 (9.3)	46 (9.3)	0.946 (0.612–1.461)	0.80	
	HCS	60 (6.7)	79 (5.7)	33 (6.7)	172 (6.2)	0.974 (0.779–1.217)	0.81	
	LBC1921	8 (4.5)	13 (5.2)	3 (3.3)	24 (4.6)	0.921 (0.511–1.661)	0.78	
	NCDS	38 (1.7)	66 (1.9)	21 (1.5)	125 (1.8)	0.971 (0.755–1.249)	0.82	
	NSHD	28 (3.6)	34 (2.7)	13 (2.5)	75 (2.9)	0.812 (0.583–1.131)	0.22	
	Whitehall II	100 (6.8)	126 (5.7)	49 (5.9)	275 (6.1)	0.907 (0.761–1.081)	0.27	
	Pooled	[421/7750]	[592/12 127]	[243/4702]	[1256/24 579]	0.963 (0.887–1.045)	0.36	
Stroke	Boyd Orr	5 (3.8)	10 (5.4)	3 (4.4)	18 (4.7)	1.118 (0.571–2.188)	0.74	0.86
	CaPS	42 (10.3)	79 (11.5)	28 (10.5)	149 (11.0)	1.023 (0.801–1.306)	0.86	
	ELSA	69 (4.1)	92 (3.4)	35 (3.3)	196 (3.6)	0.876 (0.713–1.076)	0.21	
	HAS	6 (3.9)	6 (2.3)	3 (3.0)	15 (2.9)	0.814 (0.383–1.729)	0.59	
	HCS	44 (4.9)	42 (3.0)	26 (5.2)	112 (4.0)	0.963 (0.733–1.266)	0.79	
	Pooled	[166/3261]	[229/5235]	[95/1996]	[490/10 492]	0.943 (0.827–1.076)	0.38	

CaPS: Phase V; Boyd Orr: angina Phase II.

Het, heterogeneity; MI, myocardial infarction; CaPS, Caerphilly Prospective Study; ELSA, English Longitudinal Study of Ageing; HAS, Hertfordshire Ageing Study; HCS, Hertfordshire Cohort Study; NCDS, National Child Development Study; NSHD, National Survey of Health and Development.

* No. of participants with event (%); Pooled: [no. participants with event/total no. of participants with relevant data].

† Odds ratio per T allele.

There was no evidence in English Longitudinal Study of Ageing (ELSA) that the effects of genotype differed in individuals aged below 70 years compared with those aged at least 70 years (data not shown), except for glucose (interaction *P*-value = 0.038), although the association was not significant in either age group.

In only a small number of tests did the full genotype model represent a significantly better fit than the per allele model: AH4 score in Hertfordshire Ageing Study (HAS), grip strength in LBC1921, chair rises in ELSA, BMI in HAS and Hertfordshire Cohort Study (HCS), SBP and triglycerides in NSHD, MI in Caerphilly, total cholesterol in HCS and NCDS, and stroke in HCS. Forest plots for the meta-analyses are available in the Supporting Information.

## Discussion

We investigated relationships between SNP rs401681 in the *TERT-CLPTM1L* locus and 30 age-related phenotypes in nine UK cohorts of 25 774 older adults. To our knowledge, this is the first time that this SNP has been examined with age-related traits such as physical and cognitive capability. No associations were found between genotype and any of the investigated traits on the pooled analyses, even before correcting for multiple testing. Also, no relationship was observed between genotype and age group, and although 93% of participants in our analysis were younger than 75 years, preventing an investigation into mortality, this is consistent with an observed lack of association with longevity (Rafnar *et al.*, 2009). These findings suggest that this variant is not an important contributor to a wide range of aging traits, despite being associated with several cancers (Wang *et al.*, 2008; Rafnar *et al.*, 2009; Stacey *et al.*, 2009; Kohno *et al.*, 2010; Miki *et al.*, 2010; Petersen *et al.*, 2010).

The number of investigations into the relationships between SNPs in the locus and cancer types is growing. The findings from studies examining rs401681 have been particularly interesting, with its C allele being related to an increased risk of basal cell carcinoma [odds ratio (OR): 1.20, 95% CI: 1.13–1.27 (Stacey *et al.*, 2009)], lung [OR: 1.15, 95% CI: 1.10–1.22 (Rafnar *et al.*, 2009)], bladder [OR: 1.11, 95% CI: 1.07–1.16 (Rothman *et al.*, 2010)], prostate [OR: 1.07, 95% CI: 1.03–1.11 (Rafnar *et al.*, 2009)] and cervical [OR: 1.31, 95% CI: 1.03–1.32 (Rafnar *et al.*, 2009)] cancer. However, the same allele appears to protect against melanoma [OR: 0.86, 95% CI: 0.81–0.91 (Stacey *et al.*, 2009)] and pancreatic cancer [OR (authors’ conversion): 0.84, 95% CI: 0.79–0.90 (Petersen *et al.*, 2010)]. Meanwhile, the evidence for an association with colorectal (Rafnar *et al.*, 2009; Pooley *et al.*, 2010) and endometrial (Rafnar *et al.*, 2009; Prescott *et al.*, 2010) cancer has been mixed, and there is strong evidence against any association with breast cancer (Rafnar *et al.*, 2009; Pooley *et al.*, 2010). A summary of these associations is given in Table S3 (Supporting Information). Various hypotheses about how variants in the locus may affect cancer risk have been proposed (Baird, 2010).

While in our population-based cohorts there were only 1076 cancer cases spread across numerous different tumour types, we had good statistical power to investigate associations with several other age-related phenotypes. Sample size calculations for the quantitative traits estimated that around 4500 individuals would be required to detect a beta coefficient of 0.06 *z*-score units with 80% power at the 5% significance level. As an example, such a difference would correspond to a difference in AH4 score between the two homozygote groups of around 1.2 points, assuming a standard deviation of 10. For most of the phenotypes we had sufficient power to detect differences as small as this, allowing us to conclude that associations between rs401681 and these traits are either very small or there are no associations.

Given that the C allele is associated with an increased risk of various common cancers and that the incidence of cancers generally rises with age (Nordling, 1953; DePinho, 2000; Cancer Research UK, 2010), it is an important finding that this well-powered multicohort study found that it was not associated with poorer outcomes for other aging phenotypes. Indeed, our range of investigated traits was extensive, including measures of cognitive and physical capability, blood pressure, lung function and blood lipid levels. Additionally, subgroup analysis demonstrated that on the whole, the effects were similar for men and women and for those aged below and at least 70 years.

However, it remains possible that either telomere length or telomere maintenance functions could still influence age-related traits despite the apparent absence of association between rs401681 and age-related traits in our study. First, the SNP may not substantially influence telomere length in this age group as there are conflicting reports on the relationship with rs401681 and telomere length, with the C allele associating with shorter telomeres in an elderly set of females (Rafnar *et al.*, 2009), whereas no association was observed among adults across a wider age span in recent reports (Mirabello *et al.*, 2010; Pooley *et al.*, 2010; Prescott *et al.*, 2010). Additionally, no association with telomere length was found around the *TERT* gene in a genome-wide association study (Levy *et al.*, 2010), nor in two candidate gene studies that also considered other SNPs in the region (Mirabello *et al.*, 2010; Prescott *et al.*, 2010). This would indicate that the lack of association observed in this study is not because of the choice of common polymorphism within the *TERT-CLPTM1L* locus, although mutations in the region may influence telomere length (Diaz de Leon *et al.*, 2010). Furthermore, telomere ‘length’ commonly assessed by the Cawthon assay, which measures total telomere repeats (Baird, 2005), is a crude phenotype which may not represent aspects of individual telomeres differentially relevant in cancer vs. aging traits. Second, either telomere length or another feature of telomere function could be important to age-related traits through a mechanism distinct from that by which rs401681 influences cancer risk (Baird, 2010). Other SNPs influencing telomere length or function might be useful to further explore these possibilities, particularly given the overall telomere length variability explained by identified genetic loci so far have been low (Codd *et al.*, 2010).

## Conclusion

Despite being associated with several cancers, the results of this large, multicohort investigation into a comprehensive range of aging phenotypes in a middle- to older-aged UK population do not support the hypothesis that SNP rs401681 in the *TERT-CLPTM1L* locus influences other aging traits.

## Methods

### Study populations

The Boyd Orr cohort is a historical cohort study based on children surveyed in 1937–1939 in English and Scottish districts. Participants were followed up in 1997–1998 (Phase II) and again in 2002–2003 (Phase III), during which DNA was extracted from 728 adults. Details of the study design and the data collected have been described elsewhere (Martin *et al.*, 2005).

The Caerphilly Prospective Study (CaPS) recruited 2512 men aged between 45 and 59 years in 1979–1983 from the town of Caerphilly, South Wales, and its surrounding villages. Blood samples were collected at baseline and at each of the four follow-ups (Phase II: 1984–1988, Phase III: 1989–1993, Phase IV: 1993–1997 and Phase V: 2002–2004.) Further details are available on the cohort’s website (http://www.epi.bris.ac.uk/caerphilly/caerphillyprospectivestudy.htm).

The English Longitudinal Study of Ageing contains men and women aged 50 years and over who originally participated in the Health Survey for England in 1998, 1999 or 2001. ELSA fieldwork began in 2002–2003 (Phase I) with two-yearly follow-ups in 2004–2005 (Phase II), during which blood samples were provided by 6231 participants, 2006–2007 (Phase III) and 2008–2009 (Phase IV). Details of the cohort are available elsewhere (Marmot *et al.*, 2003).

The Hertfordshire Ageing Study comprises men and women traced in 1994–1995, the first follow-up (Phase I), from singleton live births in 1920–1930 in North Hertfordshire. A total of 717 participants attended a clinic during which DNA was extracted. A second follow-up took place in 2003–2005 (Phase II). Details of the recruitment, data collected and summaries of participant characteristics have been described elsewhere (Syddall *et al.*, 2009).

The Hertfordshire Cohort Study is a younger and larger cohort, with 2997 participants born in 1931–1939 and registered with a General Practitioner in East, North and West Hertfordshire attending a clinic in 1994–2004 (Phase I). A second assessment took place in 2004–2005 for participants in East Hertfordshire (Phase II). Further details of study design, data collected and summaries of participant characteristics are available (Syddall, 2005).

The Lothian Birth Cohort 1921 Study (LBC1921) participants were all born in 1921 and completed an IQ assessment age 11. In 1999–2001 (Phase I), 550 79-year-olds, living in and around Edinburgh, attended a clinic, and in 2003–2005 (Phase II) 321 returned at 83 years old. Details of the recruitment into the study are available on its website (http://www.lothianbirthcohort.ed.ac.uk) and elsewhere (Deary *et al.*, 2004; Gow *et al.*, 2008).

The National Child Development Study (NCDS) follows individuals from all births in England, Scotland and Wales during 1 week in March 1958. In 2002–2004, a Biomedical Survey was conducted during home visits by a research nurse. DNA was extracted from 8017 participants aged 44–45 years; the sample with immortalized cell line culture (*n* = 7526) is used here. In 2008–2009, an eighth sweep was carried out during which cognitive performance tests were conducted. Further details of the study are available (Power & Elliott, 2006).

The Medical Research Council National Survey of Health and Development (NSHD) comprises participants sampled from all births in a week in March 1946 and followed up since. In 1999, at age 53 years, men and women were visited by a research nurse and consent for DNA extraction was given by approximately 2900 members of the cohort. Details of the data collected and the several phases of the study are available on the cohort’s website http://www.nshd.mrc.ac.uk and elsewhere (Wadsworth *et al.*, 2006).

The Whitehall II study targeted all civil servants aged between 35 and 55 years working in London in 1985–1988. In 2002–2004 (Phase VII), the genetics study was established and DNA was extracted from 6156 participants. Details of the study design and data collected have been described (Marmot & Brunner, 2005).

### Genotyping and quality control

Genotyping for SNP rs401681 for all cohorts, except LBC1921, was carried out by KBioscience (http://www.kbioscience.co.uk). Genotype information from LBC1921 came from a genome-wide scan performed on the Illumina Human610-Quadv1 Chip (http://www.illumina.com) (Houlihan *et al.*, 2010). Genotypic data quality was reviewed by assessing departure from Hardy–Weinberg equilibrium (HWE), clustering quality (using KBioscience software SNPviewer on their data) and call rates.

### Phenotypes

#### Cognitive capability

A number of cognitive performance tests in the different studies were used to assess cognitive capability, the capacity to undertake the mental tasks of daily living. The National Adult Reading Test (Nelson & Willison, 1991) (NART) is a widely used assessment of crystallized intelligence, i.e. acquired vocabulary and knowledge, which was measured in CaPS, LBC1921 and NSHD. The Alice Heim 4-I (Heim, 1970) (AH4) test was used in CaPS, HAS and Whitehall II, and assesses fluid intelligence, i.e. reasoning ability, particularly in novel situations. The Mill Hill vocabulary test (Raven, 1965) was used to measure crystallized verbal intelligence in HAS and Whitehall II. Different assessments of verbal memory were conducted: in ELSA and NCDS, a list of 10 common words were used, with participants asked to recall the list immediately and again after a delay, the mean score was used in the analysis; in NSHD, 15 words were used over three trials; in Whitehall II 20 words were used; responses in NSHD and Whitehall II were given in writing. Logical Memory from the Wechsler Memory Scale-Revised (Wechsler, 1987) was used in LBC1921. In Whitehall II, participants recalled in writing in 1 min as many words as possible beginning with ‘S’ to assess phonemic fluency, while in LBC1921 three letters ‘C’, ‘F’ and ‘L’ were used with responses given orally. Participants were asked to recall as many animals as possible within 1 min to measure semantic fluency; responses were given orally in CaPS, ELSA, NCDS and NSHD, and in writing in Whitehall II. To assess search speed (Richards *et al.*, 1999), 1-min letter searches among grids of letters were used, 600 letters in NSHD and 780 in ELSA and NCDS. Nonverbal reasoning was measured using Raven’s Standard Progressive Matrices in LBC1921 (Raven *et al.*, 1977).

#### Physical capability

A number of physical performance tests were used to assess physical capability, the capacity to undertake the physical tasks of daily living. Grip strength was measured in ELSA, HAS, HCS, LBC1921 and NSHD using electronic or hydraulic dynamometers, with the best measure used in the analysis where more than one trial was conducted. Several standing balance tests were conducted in the cohorts, with participants’ eyes open: Flamingo (Committee of Experts on Sports Research, 1993) (stopped at 30 s) in Boyd Orr, CaPS, HAS and HCS; side-by-side, semi-tandem and full tandem (Stevens *et al.*, 2008) in ELSA; a 30-s one-legged stance in NSHD. The timed get up and go test (Podsiadlo & Richardson, 1991) was carried out in Boyd Orr, CaPS, HAS and HCS and required participants to get up from a chair, walk 3 m, turn, walk back, turn and sit down. Timed walks over 2.44 m (8 feet) and 6 m were carried out in ELSA and LBC1921, respectively, with the fastest time used in the analysis where more than one trial was conducted. Timed chair rises (Csuka & McCarty, 1985) involved asking participants to rise from a chair and sit back down five times in ELSA, HAS and HCS, and 10 times in NSHD.

#### Anthropometry and biological function

Several measures of anthropometry and biological function were used, where available in the cohorts. BMI (kg m^−2^) was calculated as weight divided by height squared derived from measurements conducted at clinics or during a clinical interview in the home where available, or from self-reports. WHR was defined as waist circumference (cm) divided by hip circumference (cm). Where more than one sitting systolic, diastolic blood pressure (mmHg) or pulse rate (BPM) measurement was recorded at the clinical interview, the mean values were used in analysis. Spirometry was used to assess lung function: Forced vital capacity (L) and forced expiratory volume in 1 s (L); the highest value was used in the analyses. Blood samples were used to measure fibrinogen (g L^−1^), total, low-density lipoprotein (LDL) and high-density lipoprotein (HDL) cholesterol (mm), triglycerides (mm), fasting glucose (mm) and nonfasting glycosylated haemoglobin (HbA1c, %).

#### Health history

History of myocardial infarction (MI), angina, any diabetes and stroke were derived from self-reports and, where possible, from GP and hospital records.

#### Demographic variables

Data collected on age, sex, smoking status and physical activity were used to assess whether MAF varied by these variables within the cohorts. Where information on ethnicity was collected, non-white participants were excluded from analyses to avoid confounding from population stratification (Cordell & Clayton, 2005).

### Statistical methods

Statistical analysis was performed in stata 11.1 (StataCorp LP, College Station, Texas, USA ). Linear and logistic regression analyses were conducted on the continuous and dichotomous traits within the cohorts respectively. Additive models were used with genotypes coded as 0, 1 and 2 for the number of minor (T) alleles. Likelihood ratio tests were used to compare the fit of the additive models compared to the full genotype model. For continuous traits, the normality of the standardized residuals was inspected with distributional diagnostic plots. To improve the normality, natural log transformations were carried out on timed get up and go, timed walks and triglycerides. A power transformation of −2 was used on glucose and HbA1c. As with previous analyses (Kuh *et al.*, 2005), the reciprocal of time taken in seconds × 100 was used for chair rises. Cook’s distances (Cook, 2000) were plotted against fitted values, using a cut-off of four divided by sample size, to identify influential outliers in the continuous phenotypes. For the harmonization of continuous traits that were used to obtain pooled estimates of the genotypic effects, *z*-score units were calculated in each cohort by subtracting the cohort mean and dividing by its standard deviation. The overall mean for *z*-scores is 0 and standard deviation 1. Beta coefficients calculated on *z*-score units can be reverted to the original scale by multiplying by an appropriate standard deviation. Two-step (Riley *et al.*, 2010) meta-analyses were performed to obtain pooled genotypic effects, with the random-effects’ estimates presented in the tables. Meta-analyses were also stratified by sex, cigarette smoking status and physical activity (any vs. none, or any vigorous vs. none in ELSA and Whitehall II), chosen a priori. Meta-analyses were repeated after the removal of the identified influential data in the continuous traits, and results are reported on the complete data; unless the overall effect, the overall heterogeneity or the heterogeneity between subgroups were no longer significant, where the reporting is then from the restricted datasets. Within-study investigations were made in ELSA, the study with the widest age range, to assess whether the effects of genotype differed in individuals aged below and at least 70 years. In addition, within-study investigations were made into follow-up measures of cognitive and physical capability adjusting for the measure in an earlier phase. Reporting met the appropriate items of a recommended checklist (Stroup *et al.*, 2000). Quanto (Gauderman & Morrison, 2006) was used for power calculations using a MAF of 0.44. A two-tailed significance level of *P* < 0.05 was used.

## Supporting Information

**Figs S1–S28** Forest plots from meta-analyses.

**Table S1** Minor allele frequencies by sex and age group for all cohorts combined.

**Table S2** Anthropometry and biological function by genotype and cohort (full results).

**Table S3** Summary of the reported associations between SNP rs401681 and cancers.

As a service to our authors and readers, this journal provides supporting information supplied by the authors. Such materials are peer-reviewed and may be re-organized for online delivery, but are not copy-edited or typeset. Technical support issues arising from supporting information (other than missing files) should be addressed to the authors.
