# Supplementary material for: Absence of association of a single-nucleotide polymorphism in the TERT-CLPTM1L locus with age-related phenotypes in a large multicohort study: the HALCyon programme
Source: Aging Cell. 2011 Jun;10(3):520–32. doi: 10.1111/j.1474-9726.2011.00687.x (PMC3094481; doi:10.1111/j.1474-9726.2011.00687.x)
Supplement: Supplementary file 1 [file acel0010-0520-SD1.doc]

**Table of Contents**

|  |  |  |
| --- | --- | --- |

[**Figure 1: Meta-analysis for the Association between SNP rs401681 and NART Score** 3](#__RefHeading___Toc274059508)

[**Figure 2.a: Meta-analysis for the Association between SNP rs401681 and AH4 Score** 3](#__RefHeading___Toc274059509)

[**Figure 2.b: Meta-analysis for the Association between SNP rs401681 and AH4 Score Stratified by Sex** 4](#__RefHeading___Toc274059510)

[**Figure 3: Meta-analysis for the Association between SNP rs401681 and Mill Hill Score** 4](#__RefHeading___Toc274059511)

[**Figure 4: Meta-analysis for the Association between SNP rs401681 and Word Recall** 5](#__RefHeading___Toc274059512)

[**Figure 5: Meta-analysis for the Association between SNP rs401681 and Phonemic Fluency** 5](#__RefHeading___Toc274059513)

[**Figure 6: Meta-analysis for the Association between SNP rs401681 and Semantic Fluency** 6](#__RefHeading___Toc274059514)

[**Figure 7: Meta-analysis for the Association between SNP rs401681 and Search Speed** 6](#__RefHeading___Toc274059515)

[**Figure 8: Meta-analysis for the Association between SNP rs401681 and Grip Strength** 7](#__RefHeading___Toc274059516)

[**Figure 9: Meta-analysis for the Association between SNP rs401681 and Poor Balance** 7](#__RefHeading___Toc274059517)

[**Figure 10: Meta-analysis for the Association between SNP rs401681 and TUG/ Walk** 8](#__RefHeading___Toc274059518)

[**Figure 11: Meta-analysis for the Association between SNP rs401681 and Timed Chair Rises** 8](#__RefHeading___Toc274059519)

[**Figure 12: Meta-analysis for the Association between SNP rs401681 and BMI** 9](#__RefHeading___Toc274059520)

[**Figure 13: Meta-analysis for the Association between SNP rs401681 and WHR** 9](#__RefHeading___Toc274059521)

[**Figure 14: Meta-analysis for the Association between SNP rs401681 and SBP** 10](#__RefHeading___Toc274059522)

[**Figure 15: Meta-analysis for the Association between SNP rs401681 and DBP** 10](#__RefHeading___Toc274059523)

[**Figure 16: Meta-analysis for the Association between SNP rs401681 and Pulse Rate** 11](#__RefHeading___Toc274059524)

[**Figure 17: Meta-analysis for the Association between SNP rs401681 and FVC** 11](#__RefHeading___Toc274059525)

[**Figure 18: Meta-analysis for the Association between SNP rs401681 and FEV** 12](#__RefHeading___Toc274059526)

[**Figure 19: Meta-analysis for the Association between SNP rs401681 and Fibrinogen** 12](#__RefHeading___Toc274059527)

[**Figure 20: Meta-analysis for the Association between SNP rs401681 and Total Chol.** 13](#__RefHeading___Toc274059528)

[**Figure 21: Meta-analysis for the Association between SNP rs401681 and HDL Cholesterol** 13](#__RefHeading___Toc274059529)

[**Figure 22: Meta-analysis for the Association between SNP rs401681 and Triglycerides** 14](#__RefHeading___Toc274059530)

[**Figure 23: Meta-analysis for the Association between SNP rs401681 and LDL Cholesterol** 14](#__RefHeading___Toc274059531)

[**Figure 24: Meta-analysis for the Association between SNP rs401681 and Glucose** 15](#__RefHeading___Toc274059532)

[**Figure 25: Meta-analysis for the Association between SNP rs401681 and MI** 15](#__RefHeading___Toc274059533)

[**Figure 26: Meta-analysis for the Association between SNP rs401681 and Angina** 16](#__RefHeading___Toc274059534)

[**Figure 27: Meta-analysis for the Association between SNP rs401681 and Diabetes** 16](#__RefHeading___Toc274059535)

[**Figure 28: Meta-analysis for the Association between SNP rs401681 and Stroke** 17](#__RefHeading___Toc274059536)

**Figure 1: Meta-analysis for the Association between SNP rs401681 and NART Score**

**
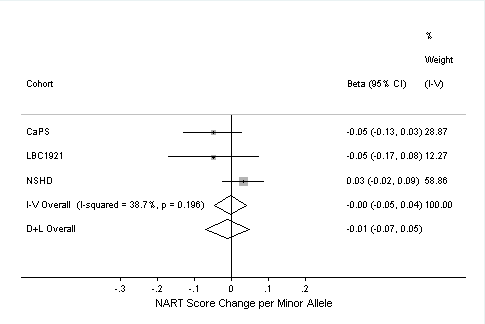
**

**Figure 2.a: Meta-analysis for the Association between SNP rs401681 and AH4 Score**

**
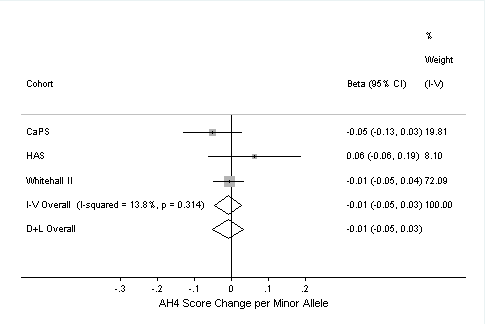
**

**Figure 2.b: Meta-analysis for the Association between SNP rs401681 and AH4 Score Stratified by Sex**

**
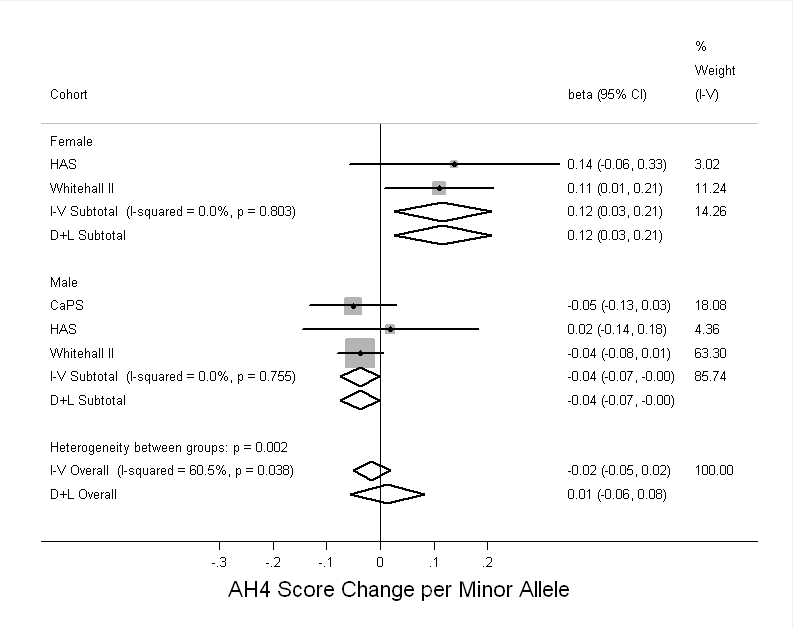
**

**Figure 3: Meta-analysis for the Association between SNP rs401681 and Mill Hill Score**

**
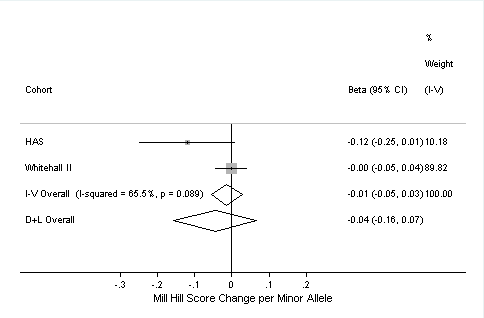
**

**Figure 4: Meta-analysis for the Association between SNP rs401681 and Word Recall**

**
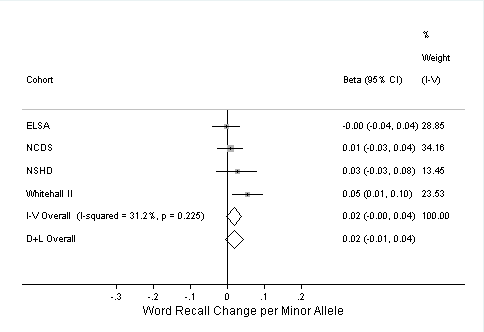
**

**Figure 5: Meta-analysis for the Association between SNP rs401681 and Phonemic Fluency**

**
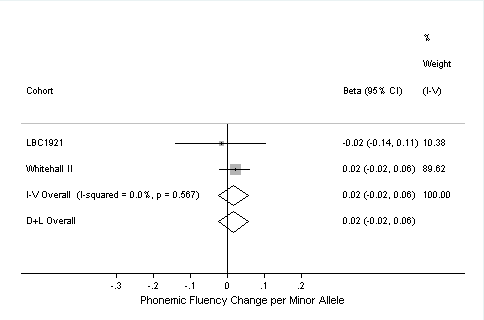
**

**Figure 6: Meta-analysis for the Association between SNP rs401681 and Semantic Fluency**

**
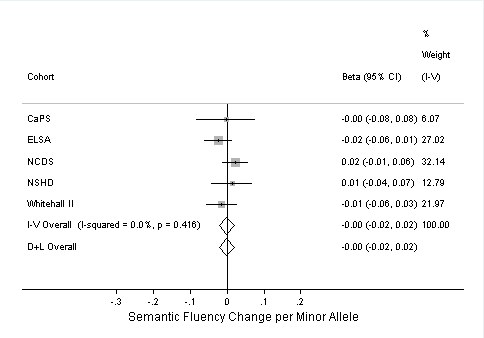
**

**Figure 7: Meta-analysis for the Association between SNP rs401681 and Search Speed**

**
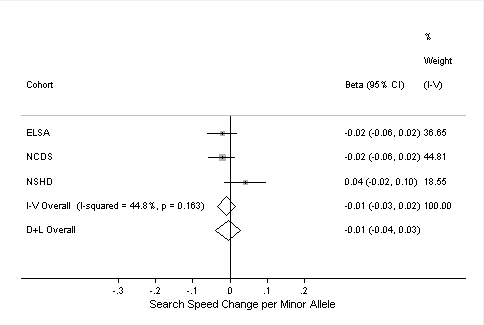
**

**Figure 8: Meta-analysis for the Association between SNP rs401681 and Grip Strength**

**
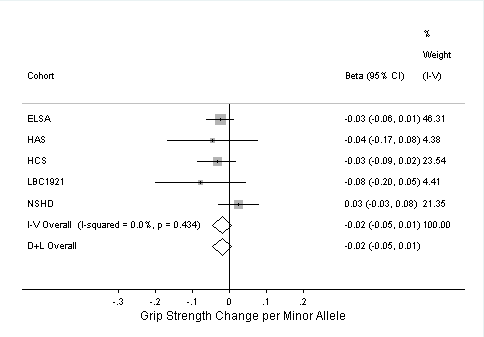
**

**Figure 9: Meta-analysis for the Association between SNP rs401681 and Poor Balance**

**
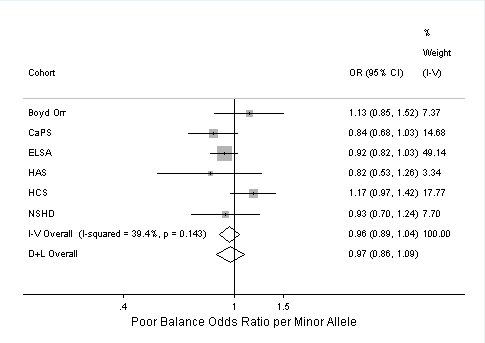
**

**Figure 10: Meta-analysis for the Association between SNP rs401681 and TUG/ Walk**

**
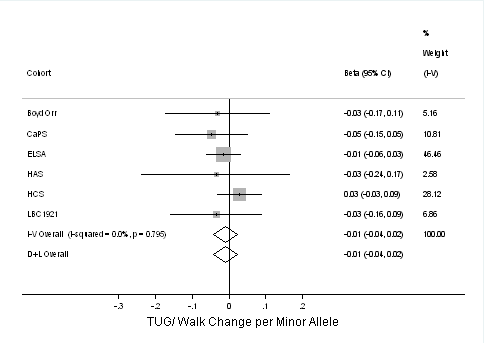
**

**Figure 11: Meta-analysis for the Association between SNP rs401681 and Timed Chair Rises**

**
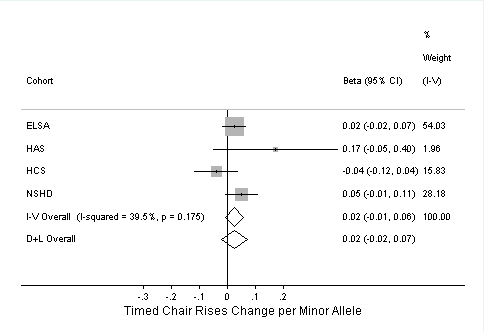
**

**Figure 12: Meta-analysis for the Association between SNP rs401681 and BMI**

**
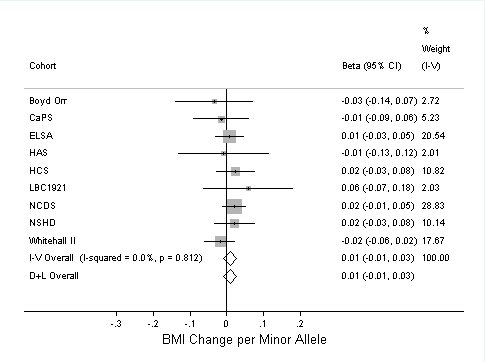
**

**Figure 13: Meta-analysis for the Association between SNP rs401681 and WHR**

**
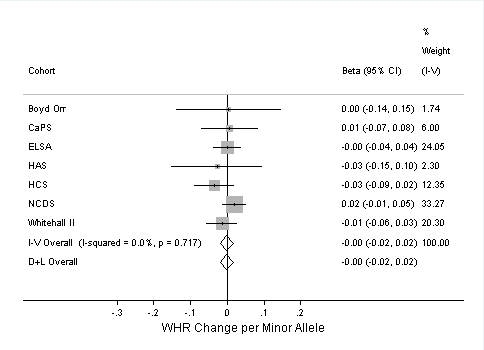
**

**Figure 14: Meta-analysis for the Association between SNP rs401681 and SBP**

**
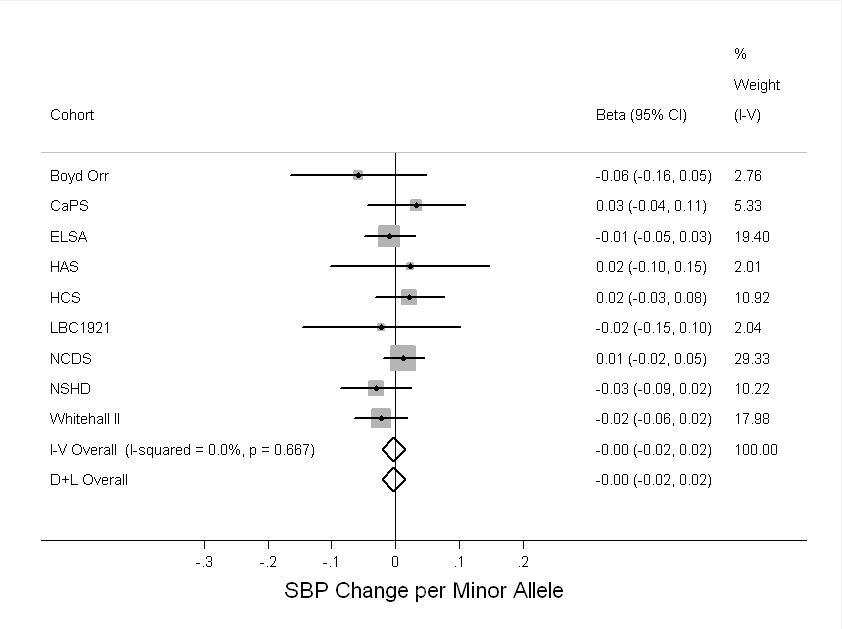
**

**Figure 15: Meta-analysis for the Association between SNP rs401681 and DBP**

**
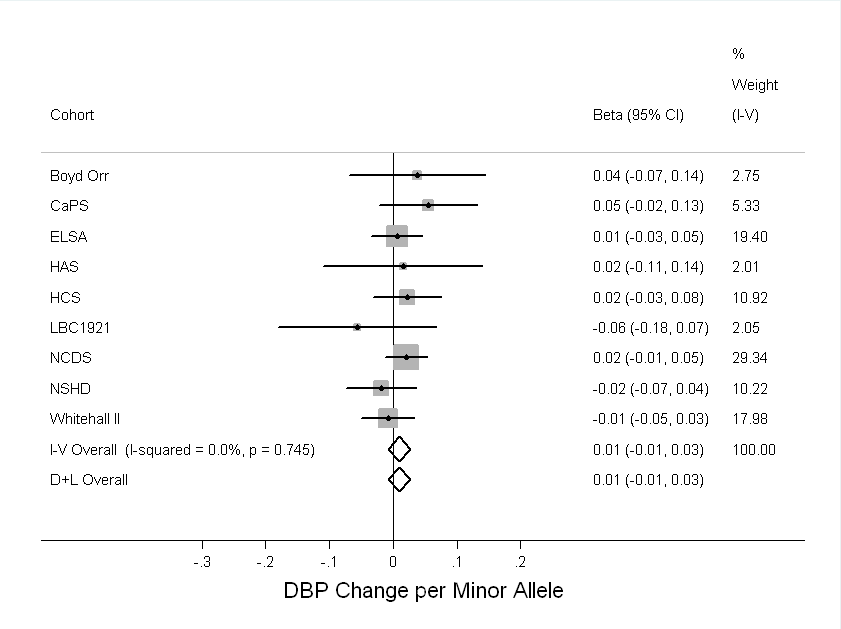
**

**Figure 16: Meta-analysis for the Association between SNP rs401681 and Pulse Rate**

**
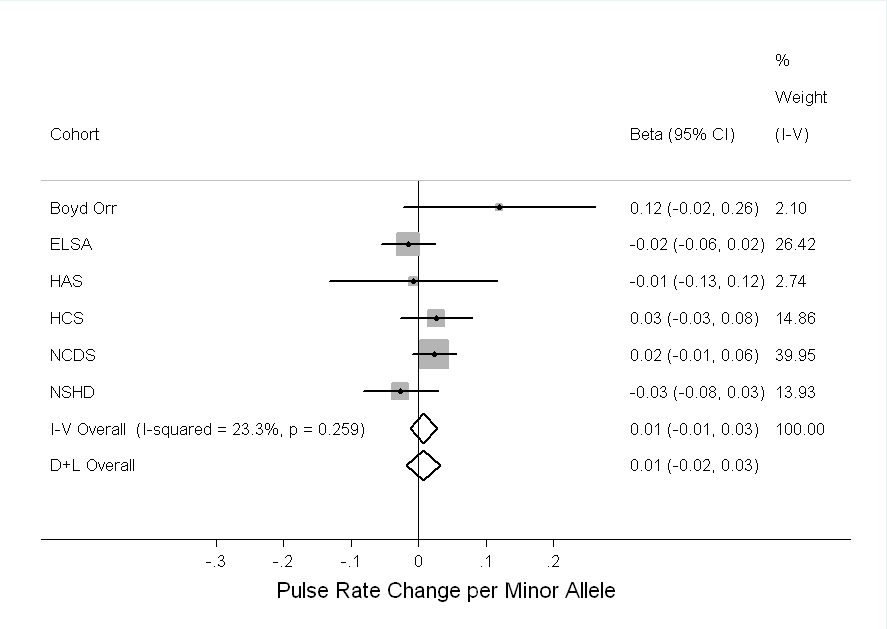
**

**Figure 17: Meta-analysis for the Association between SNP rs401681 and FVC**

**
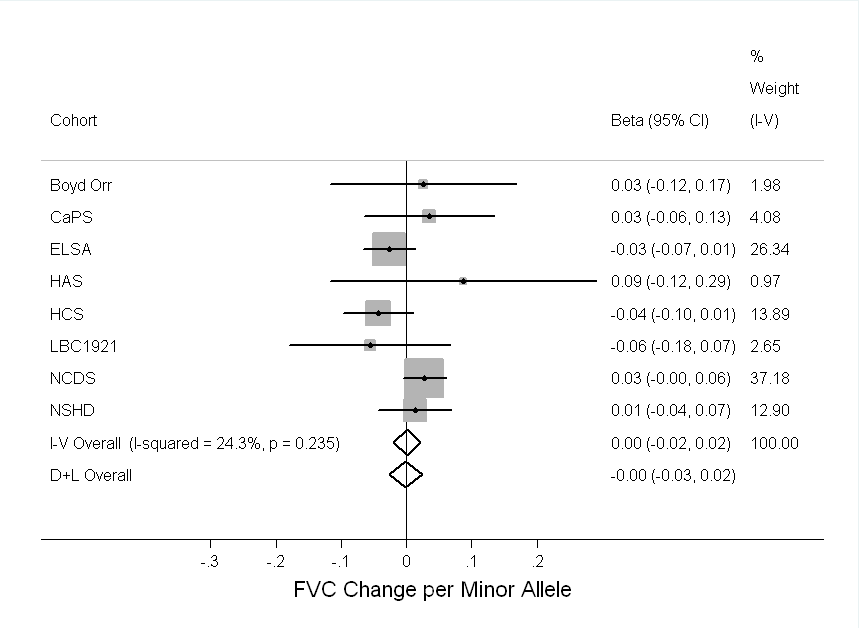
**

**Figure 18: Meta-analysis for the Association between SNP rs401681 and FEV**

**
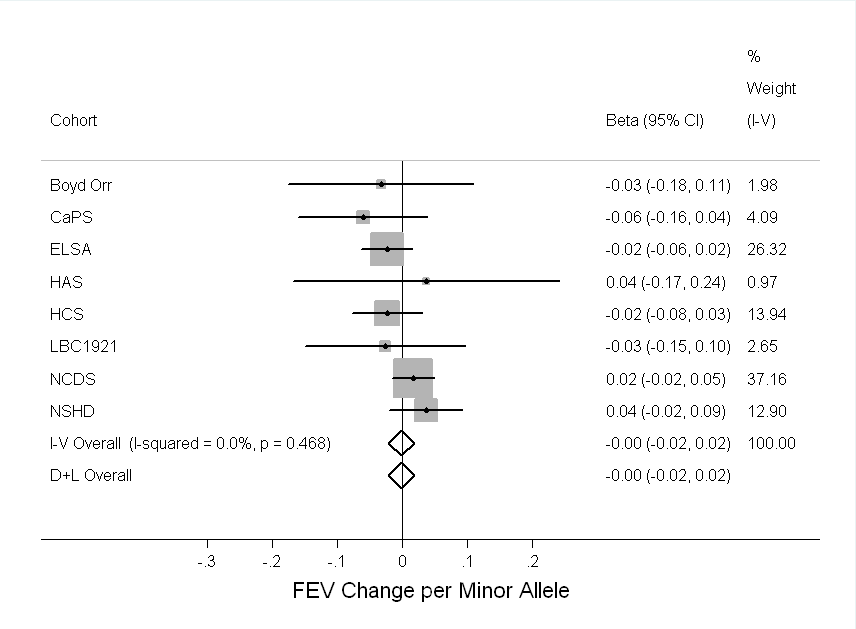
**

**Figure 19: Meta-analysis for the Association between SNP rs401681 and Fibrinogen**

**
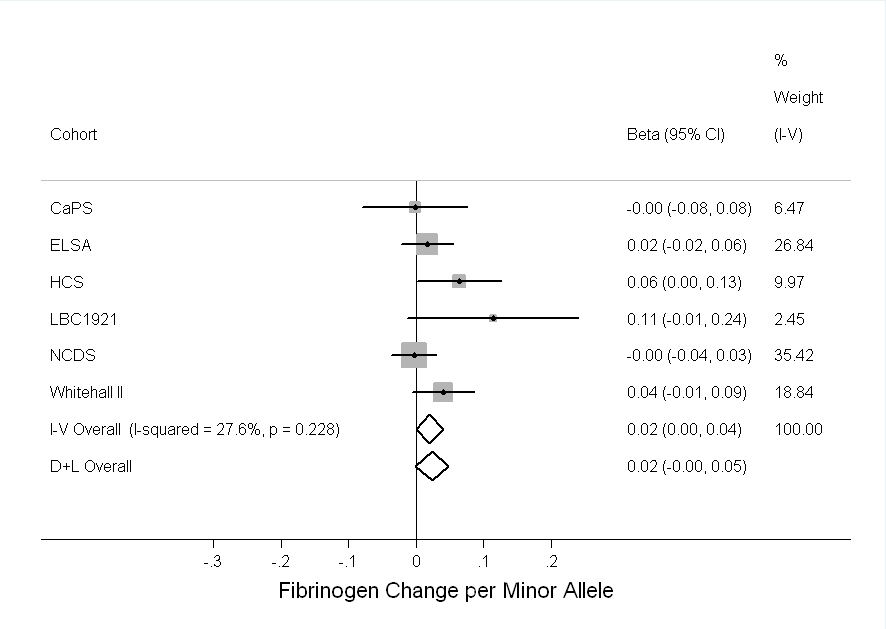
**

**Figure 20: Meta-analysis for the Association between SNP rs401681 and Total Chol.**

**
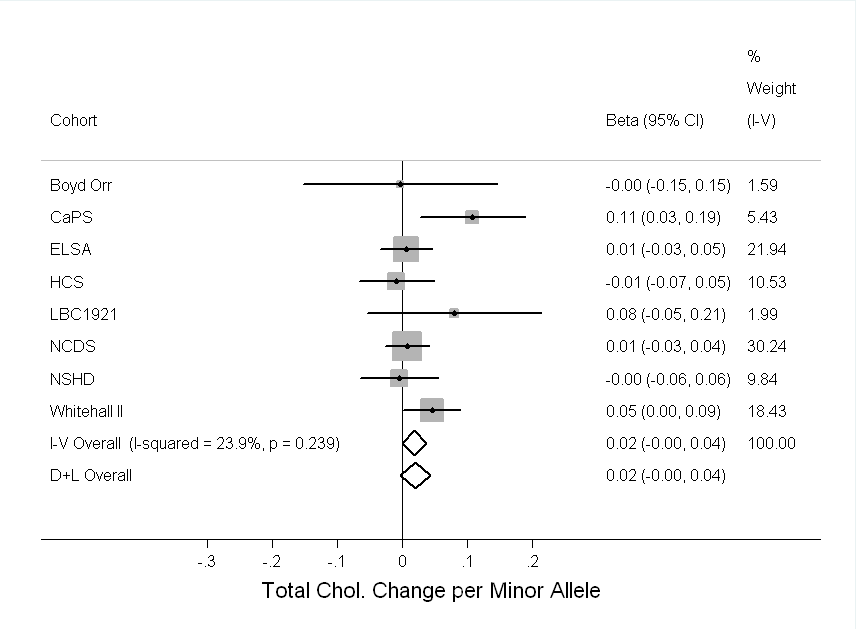
**

**Figure 21: Meta-analysis for the Association between SNP rs401681 and HDL Cholesterol**

**
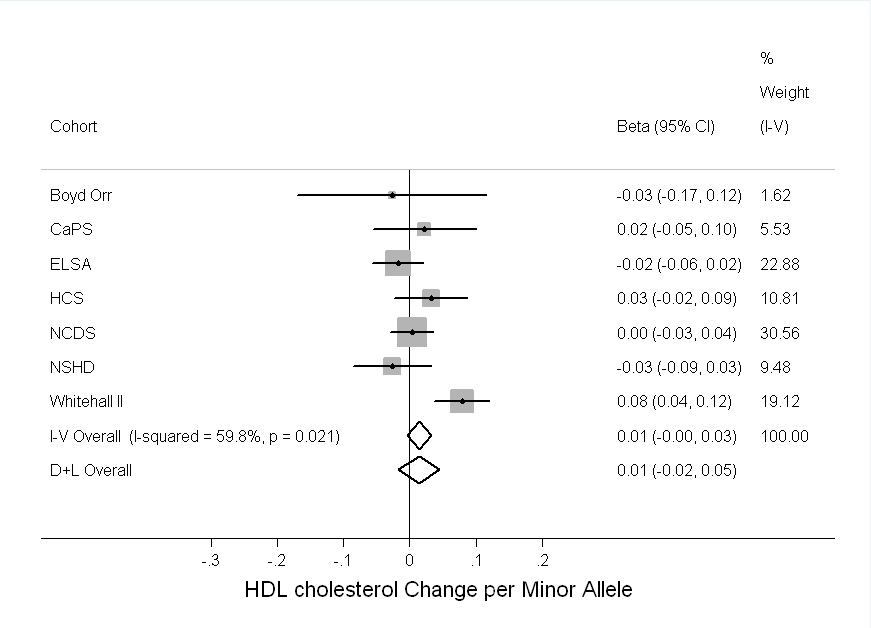
**

**Figure 22: Meta-analysis for the Association between SNP rs401681 and Triglycerides**

**
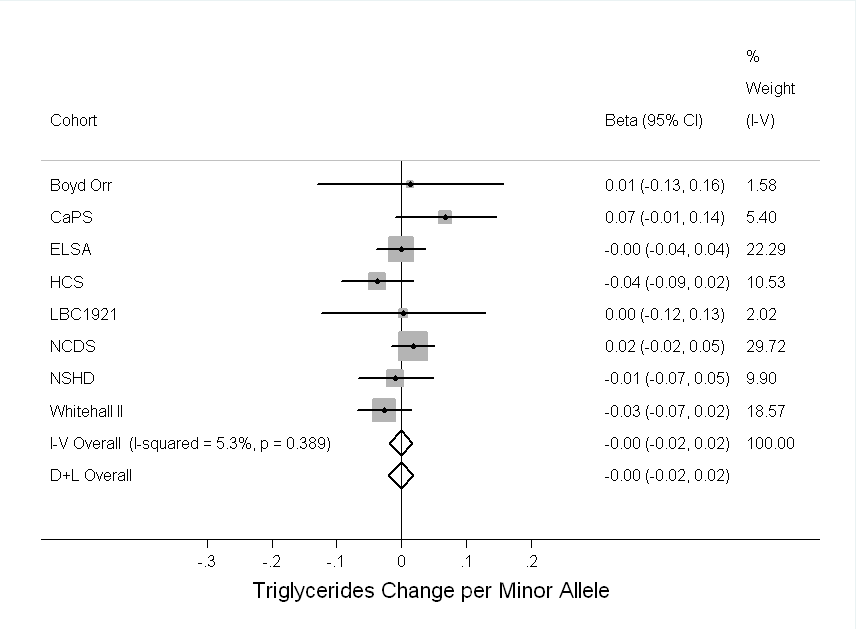
**

**Figure 23: Meta-analysis for the Association between SNP rs401681 and LDL Cholesterol**

**
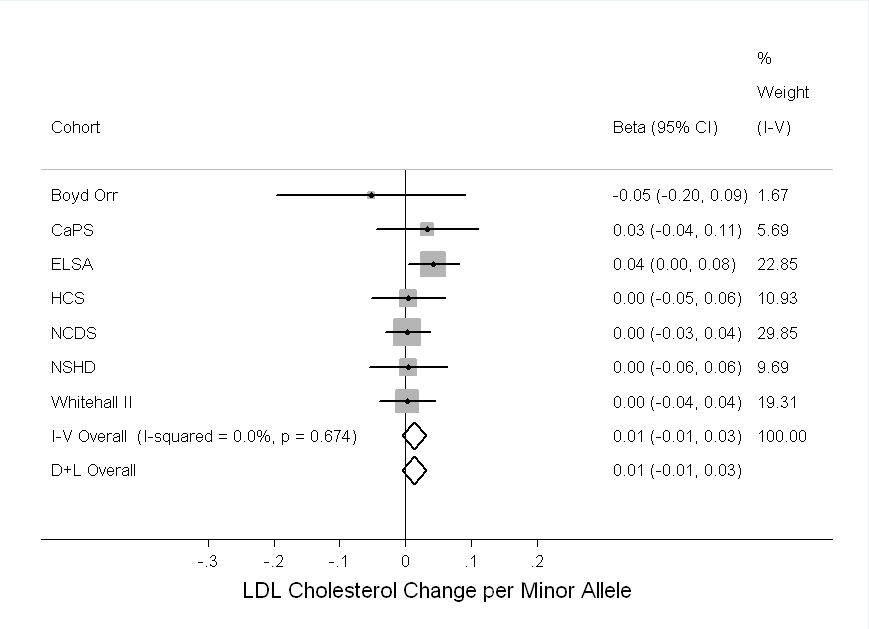
**

**Figure 24: Meta-analysis for the Association between SNP rs401681 and Glucose**

**
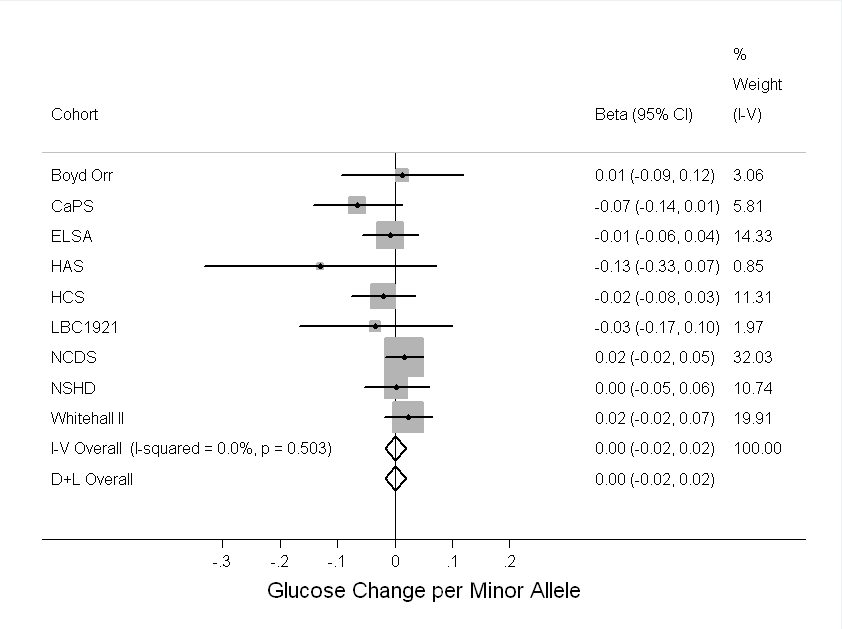
**

**Figure 25: Meta-analysis for the Association between SNP rs401681 and MI**

**
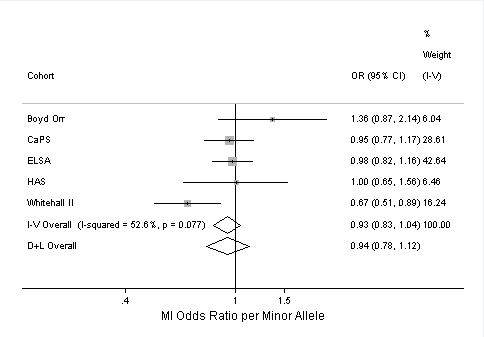
**

**Figure 26: Meta-analysis for the Association between SNP rs401681 and Angina**

**
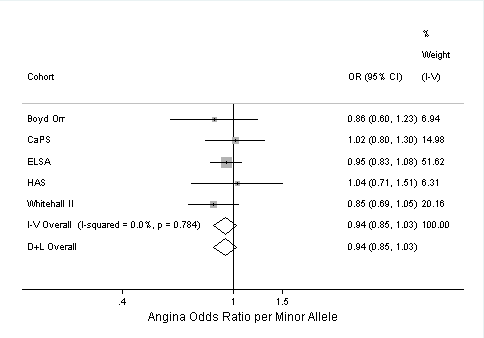
**

**Figure 27: Meta-analysis for the Association between SNP rs401681 and Diabetes**

**
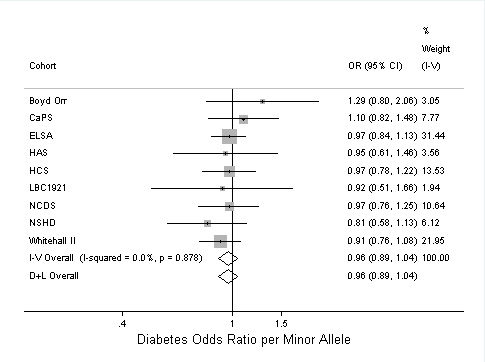
**

**Figure 28: Meta-analysis for the Association between SNP rs401681 and Stroke**

**
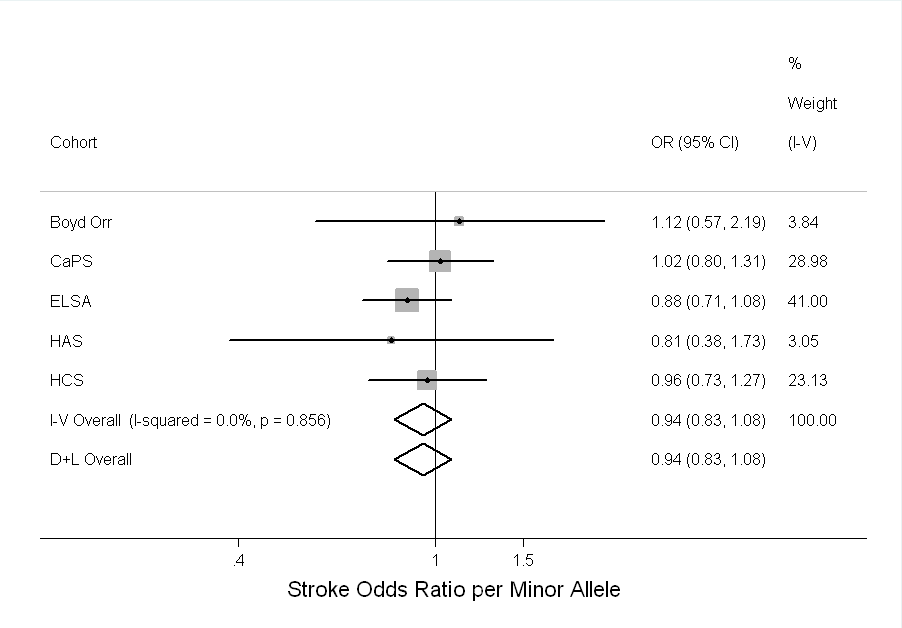
**
