# Supplementary material for: Absence of association of a single-nucleotide polymorphism in the TERT-CLPTM1L locus with age-related phenotypes in a large multicohort study: the HALCyon programme
Source: Aging Cell. 2011 Jun;10(3):520–32. doi: 10.1111/j.1474-9726.2011.00687.x (PMC3094481; doi:10.1111/j.1474-9726.2011.00687.x)
Supplement: Supplementary file 2 [file acel0010-0520-SD2.doc]

**Table S1 Minor Allele Frequencies by Sex and Age Group for All Cohorts Combined**

| Age group, | Males | Females |
| --- | --- | --- |
| years | n (MAF) | n (MAF) |
| <50 | 3736 (0.44) | 3619 (0.43) |
| 50-54 | 2642 (0.44) | 1733 (0.45) |
| 55-59 | 2147 (0.44) | 998 (0.44) |
| 60-64 | 2192 (0.43) | 1177 (0.43) |
| 65-69 | 2089 (0.43) | 1713 (0.45) |
| 70-74 | 903 (0.43) | 757 (0.44) |
| 75-79 | 480 (0.43) | 636 (0.43) |
| 80+ | 258 (0.44) | 358 (0.46) |
| Total | 14447 (0.44) | 10991 (0.44) |

**Table S2 Anthropometry and Biological Function by Genotype and Cohort (Full Results)**

| Variable | Cohort | C/C  mean (sd) [n] | C/T  mean (sd) [n] | T/T  mean (sd) [n] | Total  mean (sd) [n] | b (95% CI)^a^ | p-value | Het  p-value |
| --- | --- | --- | --- | --- | --- | --- | --- | --- |
| BMI, kg/m^2^ | Boyd Orr | 27.5 (4.0) [227] | 27.5 (4.5) [340] | 27.1 (5.0) [126] | 27.4 (4.4) [693] | -0.03 (-0.14- 0.07) | 0.52 |  |
|  | CaPS | 26.6 (3.5) [403] | 26.5 (3.7) [675] | 26.6 (3.6) [263] | 26.6 (3.6) [1341] | -0.01 (-0.09- 0.06) | 0.72 |  |
|  | ELSA | 27.7 (4.7) [1595] | 27.8 (4.7) [2588] | 27.8 (4.8) [1023] | 27.8 (4.7) [5206] | 0.01 (-0.03- 0.05) | 0.68 |  |
|  | HAS | 27.2 (4.3) [157] | 26.4 (3.7) [266] | 27.4 (4.2) [100] | 26.8 (4.0) [523] | -0.01 (-0.13- 0.12) | 0.89 |  |
|  | HCS | 27.4 (4.2) [905] | 27.2 (4.3) [1398] | 27.7 (4.5) [499] | 27.4 (4.3) [2802] | 0.02 (-0.03- 0.08) | 0.38 |  |
|  | LBC1921 | 26.0 (4.0) [177] | 26.2 (4.1) [246] | 26.5 (4.2) [90] | 26.2 (4.1) [513] | 0.06 (-0.07- 0.18) | 0.35 |  |
|  | NCDS | 27.3 (4.8) [2288] | 27.4 (4.9) [3532] | 27.5 (4.8) [1405] | 27.4 (4.9) [7225] | 0.02 (-0.01- 0.05) | 0.19 |  |
|  | NSHD | 27.3 (4.7) [769] | 27.3 (4.6) [1265] | 27.5 (4.5) [517] | 27.3 (4.6) [2551] | 0.02 (-0.03- 0.08) | 0.44 |  |
|  | Whitehall II | 26.8 (4.4) [1458] | 26.7 (4.2) [2196] | 26.7 (4.3) [828] | 26.7 (4.3) [4482] | -0.02 (-0.06- 0.02) | 0.38 |  |
|  | **Pooled** | [7979] | [12506] | [4851] | [25336] | 0.009 (-0.009- 0.026) | 0.33 | 0.81 |
| Waist-hip Ratio | Boyd Orr | 0.91 (0.09) [131] | 0.92 (0.09) [187] | 0.91 (0.09) [68] | 0.91 (0.09) [386] | 0.00 (-0.14- 0.15) | 0.96 |  |
|  | CaPS | 0.93 (0.06) [402] | 0.93 (0.06) [681] | 0.93 (0.06) [263] | 0.93 (0.06) [1346] | 0.01 (-0.07- 0.08) | 0.86 |  |
|  | ELSA | 0.89 (0.08) [1644] | 0.89 (0.08) [2636] | 0.89 (0.08) [1034] | 0.89 (0.08) [5314] | -0.00 (-0.04- 0.04) | 0.99 |  |
|  | HAS | 0.88 (0.09) [156] | 0.88 (0.09) [266] | 0.88 (0.07) [100] | 0.88 (0.08) [522] | -0.03 (-0.15- 0.10) | 0.66 |  |
|  | HCS | 0.91 (0.08) [900] | 0.91 (0.08) [1397] | 0.91 (0.08) [497] | 0.91 (0.08) [2794] | -0.03 (-0.09- 0.02) | 0.20 |  |
|  | NCDS | 0.87 (0.09) [2304] | 0.87 (0.09) [3568] | 0.87 (0.08) [1413] | 0.87 (0.09) [7285] | 0.02 (-0.01- 0.05) | 0.23 |  |
|  | Whitehall II | 0.91 (0.08) [1460] | 0.91 (0.09) [2197] | 0.91 (0.09) [830] | 0.91 (0.09) [4487] | -0.01 (-0.06- 0.03) | 0.50 |  |
|  | **Pooled** | [6997] | [10932] | [4205] | [22134] | -0.001 (-0.020- 0.018) | 0.93 | 0.72 |
| Systolic Blood Pressure, mmHg | Boyd Orr | 146.7 (21.5) [226] | 145.4 (21.0) [340] | 144.3 (20.2) [127] | 145.6 (21.0) [693] | -0.06 (-0.16- 0.05) | 0.29 |  |
|  | CaPS | 144.7 (23.0) [404] | 145.5 (22.1) [684] | 146.2 (22.7) [266] | 145.4 (22.5) [1354] | 0.03 (-0.04- 0.11) | 0.40 |  |
|  | ELSA | 135.5 (18.9) [1493] | 135.0 (18.5) [2382] | 135.3 (19.1) [954] | 135.2 (18.7) [4829] | -0.01 (-0.05- 0.03) | 0.65 |  |
|  | HAS | 155.6 (23.0) [156] | 155.4 (22.1) [263] | 156.8 (23.4) [98] | 155.7 (22.6) [517] | 0.02 (-0.10- 0.15) | 0.72 |  |
|  | HCS | 133.0 (19.5) [901] | 133.2 (18.9) [1397] | 133.9 (19.8) [500] | 133.2 (19.2) [2798] | 0.02 (-0.03- 0.08) | 0.41 |  |
|  | LBC1921 | 168.7 (28.7) [178] | 167.1 (25.4) [248] | 167.9 (26.5) [89] | 167.8 (26.7) [515] | -0.02 (-0.15- 0.10) | 0.73 |  |
|  | NCDS | 126.2 (16.5) [2304] | 126.9 (16.7) [3571] | 126.5 (15.7) [1413] | 126.6 (16.4) [7288] | 0.01 (-0.02- 0.05) | 0.44 |  |
|  | NSHD | 137.4 (20.2) [772] | 135.3 (19.9) [1254] | 136.5 (19.6) [512] | 136.1 (19.9) [2538] | -0.03 (-0.09- 0.02) | 0.28 |  |
|  | Whitehall II | 128.6 (17.1) [1466] | 128.5 (16.5) [2198] | 127.7 (16.5) [834] | 128.4 (16.7) [4498] | -0.02 (-0.06- 0.02) | 0.28 |  |
|  | **Pooled** | [7900] | [12337] | [4793] | [25030] | -0.003 (-0.020- 0.015) | 0.77 | 0.67 |
| Diastolic Blood Pressure, mmHg | Boyd Orr | 81.0 (9.8) [226] | 81.4 (10.7) [340] | 81.8 (10.9) [127] | 81.3 (10.5) [693] | 0.04 (-0.07- 0.14) | 0.49 |  |
|  | CaPS | 83.8 (11.5) [403] | 84.7 (11.5) [684] | 85.0 (11.1) [266] | 84.5 (11.4) [1353] | 0.05 (-0.02- 0.13) | 0.16 |  |
|  | ELSA | 75.2 (10.9) [1493] | 75.2 (10.8) [2382] | 75.4 (11.4) [954] | 75.2 (11.0) [4829] | 0.01 (-0.03- 0.05) | 0.77 |  |
|  | HAS | 83.3 (12.2) [156] | 83.8 (11.9) [261] | 83.6 (13.3) [98] | 83.6 (12.2) [515] | 0.02 (-0.11- 0.14) | 0.81 |  |
|  | HCS | 70.5 (11.1) [901] | 70.8 (11.3) [1397] | 71.0 (11.7) [500] | 70.7 (11.3) [2798] | 0.02 (-0.03- 0.08) | 0.41 |  |
|  | LBC1921 | 82.7 (12.0) [178] | 82.8 (13.5) [248] | 80.9 (13.4) [89] | 82.5 (13.0) [515] | -0.06 (-0.18- 0.07) | 0.37 |  |
|  | NCDS | 78.6 (10.7) [2304] | 78.9 (10.9) [3571] | 79.0 (10.4) [1413] | 78.8 (10.8) [7288] | 0.02 (-0.01- 0.05) | 0.21 |  |
|  | NSHD | 84.9 (12.3) [772] | 84.2 (11.8) [1254] | 84.5 (12.9) [512] | 84.5 (12.2) [2538] | -0.02 (-0.07- 0.04) | 0.50 |  |
|  | Whitehall II | 74.8 (10.8) [1466] | 74.8 (10.3) [2198] | 74.6 (10.5) [834] | 74.8 (10.5) [4498] | -0.01 (-0.05- 0.03) | 0.71 |  |
|  | **Pooled** | [7899] | [12335] | [4793] | [25027] | 0.009 (-0.008- 0.027) | 0.29 | 0.75 |
| Pulse Rate, BPM | Boyd Orr | 65.4 (10.8) [131] | 66.9 (11.1) [188] | 67.9 (9.5) [68] | 66.5 (10.7) [387] | 0.12 (-0.02- 0.26) | 0.10 |  |
|  | ELSA | 60.3 (15.6) [1493] | 59.8 (15.1) [2382] | 59.9 (15.1) [954] | 60.0 (15.3) [4829] | -0.02 (-0.06- 0.02) | 0.45 |  |
|  | HAS | 66.0 (12.3) [155] | 67.7 (11.1) [262] | 65.4 (10.6) [99] | 66.7 (11.4) [516] | -0.01 (-0.13- 0.12) | 0.91 |  |
|  | HCS | 68.9 (10.7) [899] | 69.2 (10.8) [1397] | 69.4 (11.1) [500] | 69.1 (10.8) [2796] | 0.03 (-0.03- 0.08) | 0.34 |  |
|  | NCDS | 71.5 (10.8) [2304] | 71.4 (10.5) [3571] | 72.0 (10.5) [1413] | 71.6 (10.6) [7288] | 0.02 (-0.01- 0.06) | 0.16 |  |
|  | NSHD | 68.4 (11.3) [772] | 67.7 (10.8) [1254] | 68.0 (10.8) [513] | 68.0 (11.0) [2539] | -0.03 (-0.08- 0.03) | 0.35 |  |
|  | **Pooled** | [5754] | [9054] | [3547] | [18355] | 0.007 (-0.018- 0.033) | 0.58 | 0.26 |
| Forced Vital Capacity, L | Boyd Orr | 3.14 (0.86) [131] | 3.19 (0.95) [185] | 3.18 (0.86) [68] | 3.17 (0.90) [384] | 0.03 (-0.12- 0.17) | 0.72 |  |
|  | CaPS | 3.26 (0.68) [236] | 3.33 (0.79) [385] | 3.30 (0.74) [160] | 3.30 (0.75) [781] | 0.03 (-0.06- 0.13) | 0.49 |  |
|  | ELSA | 3.26 (1.12) [1562] | 3.24 (1.07) [2523] | 3.21 (1.05) [1004] | 3.24 (1.08) [5089] | -0.03 (-0.07- 0.01) | 0.19 |  |
|  | HAS | 2.87 (0.69) [51] | 2.83 (0.80) [109] | 3.02 (0.88) [42] | 2.88 (0.79) [202] | 0.09 (-0.12- 0.29) | 0.41 |  |
|  | HCS | 3.45 (0.93) [887] | 3.41 (0.92) [1375] | 3.38 (0.91) [491] | 3.42 (0.92) [2753] | -0.04 (-0.10- 0.01) | 0.12 |  |
|  | LBC1921 | 2.44 (0.71) [177] | 2.38 (0.75) [246] | 2.36 (0.78) [90] | 2.40 (0.74) [513] | -0.06 (-0.18- 0.07) | 0.37 |  |
|  | NCDS | 4.18 (1.04) [2266] | 4.23 (1.05) [3492] | 4.24 (1.07) [1380] | 4.22 (1.05) [7138] | 0.03 (-0.00- 0.06) | 0.10 |  |
|  | NSHD | 3.50 (0.91) [745] | 3.49 (0.87) [1222] | 3.52 (0.92) [506] | 3.50 (0.89) [2473] | 0.01 (-0.04- 0.07) | 0.64 |  |
|  | **Pooled** | [6055] | [9537] | [3741] | [19333] | -0.001 (-0.027- 0.025) | 0.93 | 0.24 |
| Forced Expiratory Volume, L | Boyd Orr | 2.21 (0.71) [131] | 2.22 (0.73) [185] | 2.15 (0.66) [68] | 2.21 (0.71) [384] | -0.03 (-0.18- 0.11) | 0.65 |  |
|  | CaPS | 2.27 (0.55) [236] | 2.24 (0.69) [384] | 2.19 (0.61) [160] | 2.24 (0.64) [780] | -0.06 (-0.16- 0.04) | 0.23 |  |
|  | ELSA | 2.38 (0.87) [1562] | 2.36 (0.84) [2523] | 2.34 (0.84) [1004] | 2.36 (0.85) [5089] | -0.02 (-0.06- 0.02) | 0.24 |  |
|  | HAS | 2.08 (0.50) [51] | 1.98 (0.66) [109] | 2.14 (0.73) [42] | 2.04 (0.64) [202] | 0.04 (-0.17- 0.24) | 0.72 |  |
|  | HCS | 2.46 (0.70) [887] | 2.43 (0.67) [1377] | 2.43 (0.65) [495] | 2.44 (0.68) [2759] | -0.02 (-0.08- 0.03) | 0.40 |  |
|  | LBC1921 | 1.91 (0.63) [177] | 1.87 (0.62) [246] | 1.89 (0.65) [90] | 1.89 (0.63) [513] | -0.03 (-0.15- 0.10) | 0.68 |  |
|  | NCDS | 3.23 (0.85) [2266] | 3.25 (0.87) [3492] | 3.26 (0.88) [1380] | 3.25 (0.87) [7138] | 0.02 (-0.02- 0.05) | 0.31 |  |
|  | NSHD | 2.78 (0.72) [746] | 2.79 (0.68) [1222] | 2.84 (0.72) [505] | 2.80 (0.70) [2473] | 0.04 (-0.02- 0.09) | 0.20 |  |
|  | **Pooled** | [6056] | [9538] | [3744] | [19338] | -0.002 (-0.022- 0.018) | 0.86 | 0.47 |
| Fibrinogen, g/L | CaPS | 3.96 (0.81) [395] | 3.96 (0.84) [655] | 3.96 (0.86) [256] | 3.96 (0.84) [1306] | -0.00 (-0.08- 0.08) | 0.98 |  |
|  | ELSA | 3.22 (0.72) [1650] | 3.22 (0.72) [2679] | 3.25 (0.76) [1054] | 3.23 (0.73) [5383] | 0.02 (-0.02- 0.06) | 0.38 |  |
|  | HCS | 3.15 (0.85) [660] | 3.19 (0.85) [1031] | 3.26 (0.88) [362] | 3.19 (0.86) [2053] | 0.06 (0.00- 0.13) | 0.0454 |  |
|  | LBC1921 | 3.49 (0.87) [168] | 3.65 (0.90) [233] | 3.67 (0.98) [86] | 3.60 (0.90) [487] | 0.11 (-0.01- 0.24) | 0.08 |  |
|  | NCDS | 2.97 (0.63) [2235] | 2.95 (0.62) [3454] | 2.97 (0.61) [1356] | 2.96 (0.62) [7045] | -0.00 (-0.04- 0.03) | 0.87 |  |
|  | Whitehall II | 2.97 (0.62) [1216] | 3.00 (0.61) [1834] | 3.01 (0.62) [704] | 2.99 (0.61) [3754] | 0.04 (-0.01- 0.09) | 0.08 |  |
|  | **Pooled** | [6324] | [9886] | [3818] | [20028] | 0.024 (-0.001- 0.049) | 0.06 | 0.23 |
| Total Cholesterol, mmol/L | Boyd Orr | 5.63 (1.04) [125] | 5.75 (1.19) [187] | 5.56 (0.85) [59] | 5.68 (1.09) [371] | -0.00 (-0.15- 0.15) | 0.97 |  |
|  | CaPS | 5.47 (0.80) [365] | 5.59 (0.93) [656] | 5.65 (0.74) [232] | 5.57 (0.87) [1253] | 0.11 (0.03- 0.19) | 0.0088 |  |
|  | ELSA | 5.85 (0.99) [1556] | 5.90 (1.14) [2673] | 5.84 (0.90) [897] | 5.87 (1.06) [5126] | 0.01 (-0.03- 0.05) | 0.75 |  |
|  | HCS | 6.14 (0.90) [783] | 6.21 (1.10) [1314] | 6.09 (0.84) [409] | 6.17 (1.01) [2506] | -0.01 (-0.07- 0.05) | 0.78 |  |
|  | LBC1921 | 5.53 (0.91) [161] | 5.68 (1.08) [244] | 5.65 (0.80) [71] | 5.62 (0.99) [476] | 0.08 (-0.05- 0.21) | 0.24 |  |
|  | NCDS | 5.80 (0.88) [2146] | 5.86 (1.02) [3457] | 5.80 (0.80) [1238] | 5.83 (0.94) [6841] | 0.01 (-0.03- 0.04) | 0.65 |  |
|  | NSHD | 6.04 (0.88) [664] | 6.05 (1.02) [1176] | 6.03 (0.75) [424] | 6.04 (0.94) [2264] | -0.00 (-0.06- 0.06) | 0.89 |  |
|  | Whitehall II | 5.65 (0.84) [1373] | 5.70 (0.98) [2172] | 5.72 (0.73) [716] | 5.69 (0.90) [4261] | 0.05 (0.00- 0.09) | 0.0420 |  |
|  | **Pooled** | [7173] | [11879] | [4046] | [23098] | 0.020 (-0.003- 0.043) | 0.09 | 0.24 |
| HDL Cholesterol, mmol/L | Boyd Orr | 1.57 (0.46) [131] | 1.58 (0.44) [188] | 1.54 (0.38) [68] | 1.57 (0.44) [387] | -0.03 (-0.17- 0.12) | 0.71 |  |
|  | CaPS | 1.02 (0.24) [397] | 1.03 (0.25) [662] | 1.03 (0.27) [259] | 1.02 (0.25) [1318] | 0.02 (-0.06- 0.10) | 0.57 |  |
|  | ELSA | 1.53 (0.40) [1662] | 1.52 (0.38) [2694] | 1.52 (0.39) [1058] | 1.52 (0.39) [5414] | -0.02 (-0.06- 0.02) | 0.36 |  |
|  | HCS | 1.51 (0.39) [845] | 1.54 (0.44) [1323] | 1.53 (0.41) [463] | 1.52 (0.42) [2631] | 0.03 (-0.02- 0.09) | 0.26 |  |
|  | NCDS | 1.56 (0.39) [2264] | 1.56 (0.40) [3499] | 1.56 (0.39) [1387] | 1.56 (0.39) [7150] | 0.00 (-0.03- 0.04) | 0.85 |  |
|  | NSHD | 1.67 (0.51) [668] | 1.68 (0.48) [1103] | 1.64 (0.45) [452] | 1.67 (0.48) [2223] | -0.03 (-0.09- 0.03) | 0.38 |  |
|  | Whitehall II | 1.54 (0.43) [1464] | 1.58 (0.45) [2195] | 1.60 (0.49) [834] | 1.57 (0.45) [4493] | 0.08 (0.04- 0.12) | 0.0002 |  |
|  | **Pooled** | [7431] | [11664] | [4521] | [23616] | 0.014 (-0.018- 0.045) | 0.39 | 0.0207 |
| Log Triglycerides, mmol/L | Boyd Orr | 0.28 (0.44) [131] | 0.33 (0.45) [188] | 0.27 (0.43) [68] | 0.30 (0.44) [387] | 0.01 (-0.13- 0.16) | 0.86 |  |
|  | CaPS | 0.51 (0.49) [397] | 0.51 (0.52) [662] | 0.59 (0.51) [259] | 0.53 (0.51) [1318] | 0.07 (-0.01- 0.14) | 0.09 |  |
|  | ELSA | 0.45 (0.53) [1663] | 0.45 (0.50) [2695] | 0.45 (0.51) [1058] | 0.45 (0.51) [5416] | -0.00 (-0.04- 0.04) | 0.94 |  |
|  | HCS | 0.40 (0.47) [845] | 0.38 (0.47) [1323] | 0.37 (0.45) [463] | 0.38 (0.47) [2631] | -0.04 (-0.09- 0.02) | 0.19 |  |
|  | LBC1921 | 0.52 (0.39) [169] | 0.53 (0.44) [245] | 0.51 (0.37) [86] | 0.52 (0.41) [500] | 0.00 (-0.12- 0.13) | 0.97 |  |
|  | NCDS | 0.52 (0.59) [2258] | 0.54 (0.60) [3499] | 0.54 (0.60) [1386] | 0.53 (0.60) [7143] | 0.02 (-0.02- 0.05) | 0.30 |  |
|  | NSHD | 0.61 (0.60) [722] | 0.56 (0.58) [1188] | 0.61 (0.54) [481] | 0.58 (0.57) [2391] | -0.01 (-0.07- 0.05) | 0.75 |  |
|  | Whitehall II | 0.22 (0.52) [1464] | 0.20 (0.50) [2195] | 0.19 (0.51) [834] | 0.20 (0.51) [4493] | -0.03 (-0.07- 0.02) | 0.21 |  |
|  | **Pooled** | [7649] | [11995] | [4635] | [24279] | -0.001 (-0.020- 0.018) | 0.91 | 0.39 |
| LDL Cholesterol, mmol/L | Boyd Orr | 3.51 (1.09) [131] | 3.48 (1.04) [187] | 3.39 (1.04) [68] | 3.48 (1.06) [386] | -0.05 (-0.20- 0.09) | 0.47 |  |
|  | CaPS | 3.69 (0.90) [397] | 3.73 (0.90) [661] | 3.75 (0.91) [259] | 3.72 (0.90) [1317] | 0.03 (-0.04- 0.11) | 0.40 |  |
|  | ELSA | 3.55 (0.98) [1611] | 3.59 (1.00) [2631] | 3.64 (1.05) [1029] | 3.59 (1.01) [5271] | 0.04 (0.00- 0.08) | 0.0330 |  |
|  | HCS | 3.94 (0.98) [831] | 3.95 (1.01) [1306] | 3.95 (0.99) [456] | 3.95 (0.99) [2593] | 0.00 (-0.05- 0.06) | 0.88 |  |
|  | NCDS | 3.42 (0.89) [2152] | 3.43 (0.92) [3298] | 3.42 (0.91) [1318] | 3.42 (0.91) [6768] | 0.00 (-0.03- 0.04) | 0.86 |  |
|  | NSHD | 3.53 (0.99) [663] | 3.51 (0.94) [1099] | 3.54 (0.97) [451] | 3.52 (0.96) [2213] | 0.00 (-0.06- 0.06) | 0.91 |  |
|  | Whitehall II | 3.53 (0.92) [1444] | 3.51 (0.95) [2171] | 3.55 (0.97) [822] | 3.53 (0.94) [4437] | 0.00 (-0.04- 0.04) | 0.92 |  |
|  | **Pooled** | [7229] | [11353] | [4403] | [22985] | 0.013 (-0.006- 0.031) | 0.18 | 0.67 |
| HbA1c^b^ | Boyd Orr | 0.31 (0.06) [227] | 0.31 (0.06) [341] | 0.31 (0.06) [127] | 0.31 (0.06) [695] | 0.01 (-0.09- 0.12) | 0.81 |  |
| Glucose^b^ | CaPS | 0.38 (0.09) [399] | 0.38 (0.10) [663] | 0.37 (0.09) [258] | 0.38 (0.09) [1320] | -0.07 (-0.14- 0.01) | 0.10 |  |
| Glucose^b^ | ELSA | 0.42 (0.10) [994] | 0.42 (0.10) [1601] | 0.42 (0.10) [633] | 0.42 (0.10) [3228] | -0.01 (-0.06- 0.04) | 0.75 |  |
| Glucose^b^ | HAS | 0.30 (0.09) [52] | 0.30 (0.08) [108] | 0.28 (0.09) [42] | 0.29 (0.09) [202] | -0.13 (-0.33- 0.07) | 0.21 |  |
| Glucose^b^ | HCS | 0.30 (0.07) [845] | 0.30 (0.07) [1317] | 0.30 (0.07) [463] | 0.30 (0.07) [2625] | -0.02 (-0.08- 0.03) | 0.47 |  |
| HbA1c^b^ | LBC1921 | 0.32 (0.07) [149] | 0.32 (0.07) [223] | 0.31 (0.06) [80] | 0.32 (0.07) [452] | -0.03 (-0.17- 0.10) | 0.63 |  |
| HbA1c^b^ | NCDS | 0.37 (0.06) [2263] | 0.37 (0.06) [3506] | 0.37 (0.06) [1389] | 0.37 (0.06) [7158] | 0.02 (-0.02- 0.05) | 0.35 |  |
| HbA1c^b^ | NSHD | 0.32 (0.06) [725] | 0.32 (0.05) [1194] | 0.32 (0.06) [488] | 0.32 (0.06) [2407] | 0.00 (-0.05- 0.06) | 0.92 |  |
| Glucose^b^ | Whitehall II | 0.36 (0.09) [1461] | 0.36 (0.09) [2193] | 0.36 (0.08) [831] | 0.36 (0.09) [4485] | 0.02 (-0.02- 0.07) | 0.27 |  |
|  | **Pooled** | [7115] | [11146] | [4311] | [22572] | 0.001 (-0.017- 0.020) | 0.88 | 0.50 |

a: Beta coefficients per T allele based on z-scores. b: On scale 10x(glucose in mmol/L or HbA1c in %)^-2. CaPS: waist-hip ratio Phase III. HAS: FVC, FEV, HbA1c Phase II. Whitehall II: fibrinogen Phase V.

**Table S3** Summary of the reported associations between SNP rs401681 and cancers

| **Cancer** | **N**  **Cases/ Controls** | **Odds Ratio (95% CI) per C allele** | **p-value** | **Reference** |
| --- | --- | --- | --- | --- |
| Basal cell carcinoma | 2,565/ 29,405  3,468/ 38,107 | 1.25 (1.18-1.34)  1.20 (1.13-1.27) | 3.7x 10^-12^  4.8x10^-9^ | (Rafnar et al, 2009)  (Stacey et al, 2009) |
| Lung | 4,265/34,666 | 1.15 (1.10-1.22) | 7.2x10^-8^ | (Rafnar et al, 2009) |
|  | 1,952/ 1,438 | 1.15 (1.09-1.19) | 7.90x 10^-9^ | (Wang et al, 2008) |
|  | 2,343/ 1,173  1,004/1,900 | 1.14 (1.01-1.28)  1.17 (1.04-1.31) | 0.044  0.0106 | (Kohno et al, 2010)  (Miki et al, 2010) |
| Bladder | 4,147/ 34,988  3,526/ 5,117 | 1.12 (1.06–1.18)  1.11 (1.04-1.19) | 5.7 x10^-5^  2.9 x10^-3^ | (Rafnar et al, 2009)  (Rothman et al, 2010) |
| Prostate | 9,473/ 37,901 | 1.07 (1.03–1.11) | 3.6x 10^-4^ | (Rafnar et al, 2009) |
| Cervical | 276/ 28,890 | 1.31 (1.03–1.32) | 1.9x 10^-3^ | (Rafnar et al, 2009) |
| Breast | 3,645/ 30,030 | 0.98 (0.94-1.02) | 0.34 | (Rafnar et al, 2009) |
|  | 6,800/ 6,608 | 0.99 (0.94-1.04) | 0.64 | (Pooley et al, 2010) |
| Colorectal | 2,495/ 29,817 | 0.95 (0.92-0.99) | 8.4×10^-3^ | (Rafnar et al, 2009) |
|  | 2,259/ 2,246 | 0.98 (0.90-1.06) | 0.66 | (Pooley et al, 2010) |
| Melanoma | 2,443/ 30,839 | 0.88 (0.82-0.95) | 8.0×10^-4^ | (Rafnar et al, 2009) |
|  | 3,843/ 41,963 | 0.86 (0.81-0.91) | 5.0x 10^-8^ | (Stacey et al, 2009) |
|  | 782/ 999 | 1.01 (0.86-1.19) | 0.91 | (Pooley et al, 2010) |
| Endometrial | 470/ 28,890 | 1.21 (1.06-1.38) | 5.5×10^-3^ | (Rafnar et al, 2009) |
|  | 674/ 1,685 | 0.96 (0.84-1.10) | Not given | (Prescott et al, 2010) |
| Kidney | 987/ 30,722 | 1.08 (0.97-1.19) | 0.14 | (Rafnar et al, 2009) |
| Lymphoma | 248/ 28,890 | 0.87 (0.72-1.05) | 0.14 | (Rafnar et al, 2009) |
| Multiple myeloma | 126/ 28,890 | 1.21 (0.93-1.58) | 0.16 | (Rafnar et al, 2009) |
| Ovarian | 497/ 28,890 | 0.98 (0.84-1.14) | 0.80 | (Rafnar et al, 2009) |
| Pancreatic | 301/ 28,890 | 0.99 (0.81-1.21) | 0.92 | (Rafnar et al, 2009) |
|  | 3,532/ 3,642 | 0.84 (0.79-0.90) | 3.66x10^-7^ | (Petersen et al, 2010) |
| Skin SCC | 547/ 28,890 | 1.14 (1.00-1.30) | 0.051 | (Rafnar et al, 2009) |
|  | 1,103/ 35,824 | 1.04 (0.94-1.16) | 0.39 | (Stacey et al, 2009) |
| Stomach | 762/ 28,890 | 0.96 (0.86-1.07) | 0.45 | (Rafnar et al, 2009) |
| Thyroid | 528/ 28,890 | 0.97 (0.85-1.10) | 0.64 | (Rafnar et al, 2009) |
| Head and neck SCC | 1,079/ 1,115 | Not given | 0.605 | (Liu et al, 2010) |

SCC: squamous cell carcinoma

Kohno T, Kunitoh H, Shimada Y, Shiraishi K, Ishii Y, Goto K, Ohe Y, Nishiwaki Y, Kuchiba A, Yamamoto S, Hirose H, Oka A, Yanagitani N, Saito R, Inoko H & Yokota J (2010) Individuals susceptible to lung adenocarcinoma defined by combined HLA-DQA1 and TERT genotypes. *Carcinogenesis* **31:** 834-841

Liu Z, Li G, Wei S, Niu J, Wang L, Sturgis EM & Wei Q (2010) Genetic variations in TERT-CLPTM1L genes and risk of squamous cell carcinoma of the head and neck. *Carcinogenesis* **31:** 1977-1981

Miki D, Kubo M, Takahashi A, Yoon K, Kim J, Lee GK, Zo JI, Lee JS, Hosono N, Morizono T, Tsunoda T, Kamatani N, Chayama K, Takahashi T, Inazawa J, Nakamura Y & Daigo Y (2010) Variation in TP63 is associated with lung adenocarcinoma susceptibility in Japanese and Korean populations. *Nat Genet* **42:** 893-896

Petersen GM, Amundadottir L, Fuchs CS, Kraft P, Stolzenberg-Solomon RZ, Jacobs KB, Arslan AA, Bueno-de-Mesquita HB, Gallinger S, Gross M, Helzlsouer K, Holly EA, Jacobs EJ, Klein AP, LaCroix A, Li D, Mandelson MT, Olson SH, Risch HA, Zheng W et al (2010) A genome-wide association study identifies pancreatic cancer susceptibility loci on chromosomes 13q22.1, 1q32.1 and 5p15.33. *Nat Genet* **42:** 224-228

Pooley KA, Tyrer J, Shah M, Driver KE, Leyland J, Brown J, Audley T, McGuffog L, Ponder BAJ, Pharoah PDP, Easton DF & Dunning AM (2010) No Association between TERT-CLPTM1L Single Nucleotide Polymorphism rs401681 and Mean Telomere Length or Cancer Risk. *Cancer Epidemiol Biomarkers Prev* **19:** 1862-1865

Prescott J, McGrath M, Lee I, Buring JE & De Vivo I (2010) Telomere length and genetic analyses in population-based studies of endometrial cancer risk. *Cancer* **116:** 4275-4282

Rafnar T, Sulem P, Stacey SN, Geller F, Gudmundsson J, Sigurdsson A, Jakobsdottir M, Helgadottir H, Thorlacius S, Aben KKH, Blondal T, Thorgeirsson TE, Thorleifsson G, Kristjansson K, Thorisdottir K, Ragnarsson R, Sigurgeirsson B, Skuladottir H, Gudbjartsson T, Isaksson HJ et al (2009) Sequence variants at the TERT-CLPTM1L locus associate with many cancer types. *Nat Genet* **41:** 221-227

Rothman N, Garcia-Closas M, Chatterjee N, Malats N, Wu X, Figueroa JD, Real FX, Van Den Berg D, Matullo G, Baris D, Thun M, Kiemeney LA, Vineis P, De Vivo I, Albanes D, Purdue MP, Rafnar T, Hildebrandt MAT, Kiltie AE, Cussenot O et al (2010) A multi-stage genome-wide association study of bladder cancer identifies multiple susceptibility loci. *Nat. Genet* **42:** 978-984

Stacey SN, Sulem P, Masson G, Gudjonsson SA, Thorleifsson G, Jakobsdottir M, Sigurdsson A, Gudbjartsson DF, Sigurgeirsson B, Benediktsdottir KR, Thorisdottir K, Ragnarsson R, Scherer D, Hemminki K, Rudnai P, Gurzau E, Koppova K, Botella-Estrada R, Soriano V, Juberias P et al (2009) New common variants affecting susceptibility to basal cell carcinoma. *Nat. Genet* **41:** 909-914

Wang Y, Broderick P, Webb E, Wu X, Vijayakrishnan J, Matakidou A, Qureshi M, Dong Q, Gu X, Chen WV, Spitz MR, Eisen T, Amos CI & Houlston RS (2008) Common 5p15.33 and 6p21.33 variants influence lung cancer risk. *Nat. Genet* **40:** 1407-1409
